# Modification of SnO_2_ Electron Transport Layer in Perovskite Solar Cells

**DOI:** 10.3390/nano12234326

**Published:** 2022-12-05

**Authors:** Helen Hejin Park

**Affiliations:** 1 Advanced Materials Division, Korea Research Institute of Chemical Technology (KRICT), Daejeon 34114, Republic of Korea; hhpark@krict.re.kr; 2 Department of Advanced Materials and Chemical Engineering, University of Science and Technology (UST), Daejeon 34113, Republic of Korea

**Keywords:** metal-halide perovskite, photovoltaics, solar-cell materials, tin-oxide-electron- transport layers

## Abstract

Rapid development of the device performance of organic-inorganic lead halide perovskite solar cells (PSCs) are emerging as a promising photovoltaic technology. Current world-record efficiency of PSCs is based on tin oxide (SnO_2_) electron transport layers (ETLs), which are capable of being processed at low temperatures and possess high carrier mobilities with appropriate energy- band alignment and high optical transmittance. Modification of SnO_2_ has been intensely investigated by various approaches to tailor its conductivity, band alignment, defects, morphology, and interface properties. This review article organizes recent developments of modifying SnO_2_ ETLs to PSC advancement using surface and bulk modifications, while concentrating on photovoltaic (PV) device performance and long-term stability. Future outlooks for SnO_2_ ETLs in PSC research and obstacles remaining for commercialization are also discussed.

## 1. Introduction

Solar energy is one of the most plentiful energy resources accessible to humankind. Among the various PV technologies, currently monocrystalline-silicon-solar cells dominate the PV market due to its high PCE of 26.1% and high working stability, but suffers from cost-intensive production cost of the highly purified monocrystalline silicon. The low production cost and rapid increase in unit-cell efficiency of organic-inorganic PSCs, which is currently 25.7% [1], enables it to compete with silicon-solar cells. Organometallic- halide perovskites are based on the chemical formula of *ABX*_3_, where *A* is organic or metal cations, such as methylammonium (CH_3_NH_3_^+^ (MA^+^)), formamidinium ((NH_2_)_2_CH^+^ (FA^+^)), Rb^+^, or Cs^+^, *B* is metal ions, such as Pb^2+^ or Sn^2+^, and *X* is halogen ions, such as I^−^, Br^−^, or Cl^−^. Organometallic-halide perovskites possess features of an ideal absorber material, including high absorption coefficients (~10^−4^ cm^−1^), long carrier-diffusion lengths (>1 μm), ambipolar-charge-transport capabilities, and low exciton-binding energy (20–50 meV), [2].

A typical *n*-*i*-*p* PSC device structure consists of glass/transparent conducting oxide (TCO)/*n*-type ETL/perovskite absorber/*p*-type hole transport layer (HTL)/metal contact. The bottom TCO is usually fluorine-doped tin oxide (FTO) or indium tin oxide (ITO), and the top metal contact is typically gold (Au), silver (Ag), or aluminum (Al). For a typical *p*-*i*-*n* PSC device, the structure consists of glass/TCO/HTL/perovskite/ETL/top metal contact.

Among the various layers in PSCs, the ETL plays a key role in photovoltaic performance and the charge dynamics. Traditionally, mesoporous titanium dioxide (*mp*-TiO_2_) was used as the ETL in *n*-*i*-*p* structured devices with 2,2′,7,7′-tetrakis-(*N*,*N*-di-4-methoxyphenylamino)-9,9′-spirobifluorene (spiro-OMeTAD) or poly[bis(4-phenyl)(2,5,6-trimethylphenyl)amine] (PTAA) as the HTL. However, high-temperature sintering is required for the *mp*-TiO_2_ to enhance the crystallinity and remove organic material in the TiO_2_ paste. Furthermore, there is a charge barrier at the TiO_2_/perovskite interface, leading to inefficient charge transfer and a large charge accumulation at the interface. Other organic materials, including fullerene and its derivatives, have also been used as ETLs in PSCs [3,4]. However, such organic-conducting materials have unreliable stability against light, thermal, and environmental factors. Other low-temperature processed metal oxides, such as tin oxide (SnO_2_) [5], zinc oxide (ZnO) [6,7], tungsten oxide (WO_3_) [8], indium oxide (In_2_O_3_) [9], niobium oxide (Nb_2_O_5_) [10], cerium oxide (CeO*_x_*) [11], Zn_2_SO_4_ [12], BaSnO_3_ [13], SrTiO_3_ [14], and cadmium sulfide (CdS) [15] have also been explored. ZnO can be processed at low temperatures, but the -OH residue on the ZnO surface causes to decompose the perovskite layer leading to environmentally unstable devices. The high toxicity of cadmium becomes a concern regarding to CdS. In addition, the low bandgap of CdS (2.4 eV) causes current loss in the UV range.

Compared to the traditional *mp*-TiO_2_ ETL in PSCs, SnO_2_ can be fabricated at lower temperatures, which expands its possible applications to flexible substrates. SnO_2_ also has a more favorable band alignment in PSCs compared to TiO_2_, as the conduction-band minimum is lower than that of TiO_2_, which induces higher efficiency of charge injection. It also possesses good electrical conductivity and high optical transmittance [16,17]. Furthermore, the larger bandgap of SnO_2_ compared to TiO_2_ makes it less vulnerable to UV light. Thus, the device stability can be improved by suppressed UV-related photochemical reaction. Some drawbacks of SnO_2_ are the bulk and surface defects in the pristine films, such as surface-adsorbed hydroxyl groups, uncoordinated Sn^4+^, and oxygen vacancies, which deteriorate electronic properties and capture electrons, degrading the stability and efficiency of PSCs. Such drawbacks of SnO_2_ can be alleviated by modifying the bulk or surface of the film, which will be discussed in this review.

Suitable ETL materials should (i) have a favorable energy-band alignment with the absorber layer for efficient electron transfer from the perovskite to ETL, (ii) exhibit high transparency to minimize optical losses, (iii) possess a wide optical bandgap so that there will be no contribution as a second absorber layer and can also benefit the photochemical reaction, as the wide bandgap will prevent reaction with high-energy radiation, such as UV-light, which is key for the device stability, (iv) have a high conductivity for high fill factors and low series resistance, (v) exhibit appropriate hydrophobic behavior to tolerate long-term exposure to humidity, (vi) be environmentally friendly, and (vii) have low fabrications and materials costs [17,18,19,20,21].

The modification of SnO_2_ has been investigated by various institutions and has contributed to enhancing the PV performance and device stability of PSCs. In this review, the recent contributions of SnO_2_ ETLs in PSCs are organized based on photovoltaic performance and stability. Section 2 will cover modification of SnO_2_ ETLs in PSCs using elemental doping, insertion of metal-oxide layers, ionic compounds, carbon materials, and organic molecules. Section 2 will also discuss the long-term stability of the modified SnO_2_ ETL-based PSCs. Development in the device efficiency and stability are organized in Table 1, Table 2, Table 3, Table 4, Table 5, Table 6, Table 7, Table 8 and Table 9, respectively. Section 3 will summarize the SnO_2_ modification approaches and discuss future outlooks for commercialization.

## 2. Device Performance and Stability of SnO_2_ ETL-Based PSCs

### 2.1. Elemental Doping

Elemental doping of SnO_2_ is a straightforward method to effectively alter the conductivity, defect states, and energy level. SnO_2_ can be simply doped by various elements to tune its electrical and chemical properties. SnO_2_ is an *n*-type material. The tetravalent- Sn sites can be replaced by cations with low valence states, such as gallium (Ga^3+^), cobalt (Co^3+^), zinc (Zn^2+^), magnesium (Mg^2+^), and lithium (Li^+^) for *p*-type doping, or can be substituted by cations with high valence states, such as antimony (Sb^5+^), molybdenum (Mo^5+^), tantalum (Ta^5+^), and niobium (Nb^5+^) for *n*-type doping. 

**Figure 1:**
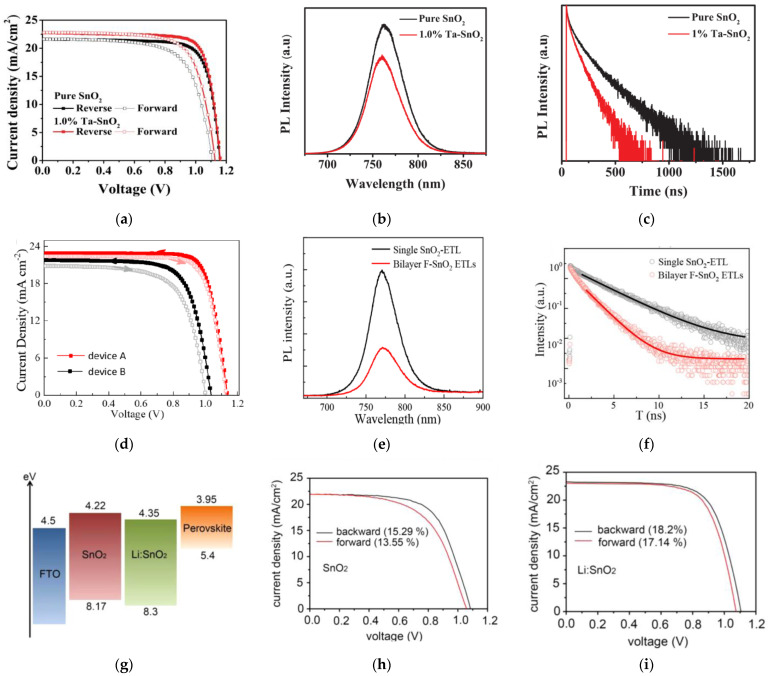
(**a**) Illuminated current density vs. voltage (*J*-*V*) curves under reverse and forward voltage scanning of PSC devices, (**b**) photoluminescence (PL) spectra, and (**c**) time-resolved PL (TRPL) spectra of perovskite films on undoped and Ta-doped SnO_2_ ETLs. Reproduced from [22] with permission from AIP Publishing, 2019. (**d**) Illuminated *J*-*V* curves under 1 SUN under reverse and forward voltage scanning of PSC devices, (**e**) photoluminescence (PL) spectra, and (**f**) TRPL spectra of perovskite films on undoped and bilayer F-doped SnO_2_ ETLs. Reproduced from [23] with permission from American Chemical Society (ACS) Publications, 2018. (**g**) Energy diagram of FTO, ETLs, and perovskite. Illuminated *J*-*V* curve of PSC based on (**h**) undoped SnO_2_ and (**i**) Li-doped SnO_2_ under 1 SUN. Reproduced from [24] with permission from Elsevier, 2016.

Li et al. reported a significant improvement in conductivity without declining the transmittance by doping SnO_2_ with Ta [22]. Ta-doped SnO_2_ improved the PCE from 19.5% to 20.9% by improved fill factor (*FF*) and short-circuit current density (*J_SC_*), as shown in the illuminated current-density (*J*-*V*) curves in Figure 1a. This is due to effective acceleration of electron collection and transfer, and reduction in recombination at the ETL/absorber interface, and shown in the steady state photoluminescence (PL) and time-resolved PL (TRPL) results in Figure 1b,c.

Doping can also be performed by non-metallic elements, such as fluorine (F^−^), to replace the oxygen anion sites of SnO_2_. X. Gong et al. reported that gradual bilayer replacement of F^−^ into SnO_2_ can decrease the band offset and the ETL/perovskite interface, resulting in increased built-in electric field and enhancing the open-circuit voltage (*V_OC_*) [23] from 1.03 to 1.13 V. PSC devices resulted in enhancement of PCE from 16.3% to 20.2%, as shown in Figure 1d. Improved electron-extraction ability is suggested, based on the steady-state PL and TRPL results in Figure 1e,f. Encapsulated devices retained over 85% of its initial efficiency after stored in air at room temperature and 40–50% relative humidity for 300 h.

M. Park et al. reported a solution process to effectively dope SnO_2_ with Li at a low processing temperature [24]. Li-doping enhanced the conductivity of SnO_2_ and produced a reduction of the conduction-band energy, as shown in Figure 1g, facilitating the transfer of electrons and reduced the charge recombination. This resulted in improved *V_OC_*, *FF*, and *J_SC_* with a PCE increase of 15.3% to 18.2%, as shown in Figure 1h,i.

Niobium doping of SnO_2_ was reported by Ren et al. using a solution-processable low-temperature method [25]. The improvement in PV performance originates from the increased conductivity and improved surface morphology, which lead to enhanced electron extraction and inhibited charge recombination. Unencapsulated devices maintained 90% of its initial PCE after 288 h stored in air at room temperature.

Yttrium (Y^3+^) doping of SnO_2_ reported by Yang et al. promotes more homogeneous distribution and well-aligned SnO_2_ nanosheet arrays, which leads to improved electron transfer from the absorber to the ETL [26]. Enlarged bandgaps from the Y-doping and a higher conduction-band energy allows improved energy band alignment and reduced the charge recombination at the ETL/absorber interface. This improved the PCE of the PSC from 13.4% to 17.3% by increasing *V_OC_*, *FF*, and *J_SC_*.

Bai et al. reported that Sb:SnO_2_ nanocrystals was used to replace the undoped SnO_2_ ETL [27]. This shifted the Fermi-energy level upward, which improved the energy- band alignment and reduced charge recombination. Electron = recombination lifetime was longer, and *V_OC_* and *FF* increased with less photocurrent hysteresis. PCE values increased from 15.7% to 17.2%. Unsealed devices retained over 95% of its initial efficiency after 504 h stored in a desiccator at room temperature.

Gallium-doped SnO_2_ reported by Roose et al. observed decreased trap-state density in the ETL, leading to a reduced recombination rate [28]. *V_OC_* and *FF* increased from 1.00 to 1.07 V and 57.0% to 70.0%, respectively, resulting in a PCE enhancement of 12.5% to 17.0%. Unencapsulated devices maintained about 70% of its initial efficiency after 1000 h under continuous 1 SUN illumination in nitrogen (N_2_).

Xiong et al. reported Mg-doped SnO_2_ as the ETL in PSCs [29]. An optimum-Mg content resulted in uniform, smooth, and dense films with reduced free-electron density, which suppressed the charge recombination, and increased electron mobility, which enabled fast extraction of electrons, contributing to improved *J_SC_*. PCE improved from 8.2% to 15.2% by doping the ETL with Mg. Unencapsulated devices maintained over 90% of its initial PCE after 720 h of storage in air with <20% relative humidity.

Aluminum doped SnO_2_ reported by Chen et al. resulted in increased *J_SC_* and *FF* by using a low-temperature solution-processable method [30]. Doping SnO_2_ with Al enhanced the charge transport and electron extraction based on TRPL tests. PSC devices exhibited improved PCE of 9.0% to 12.1%.

### 2.2. Metal Oxide

**Figure 2:**
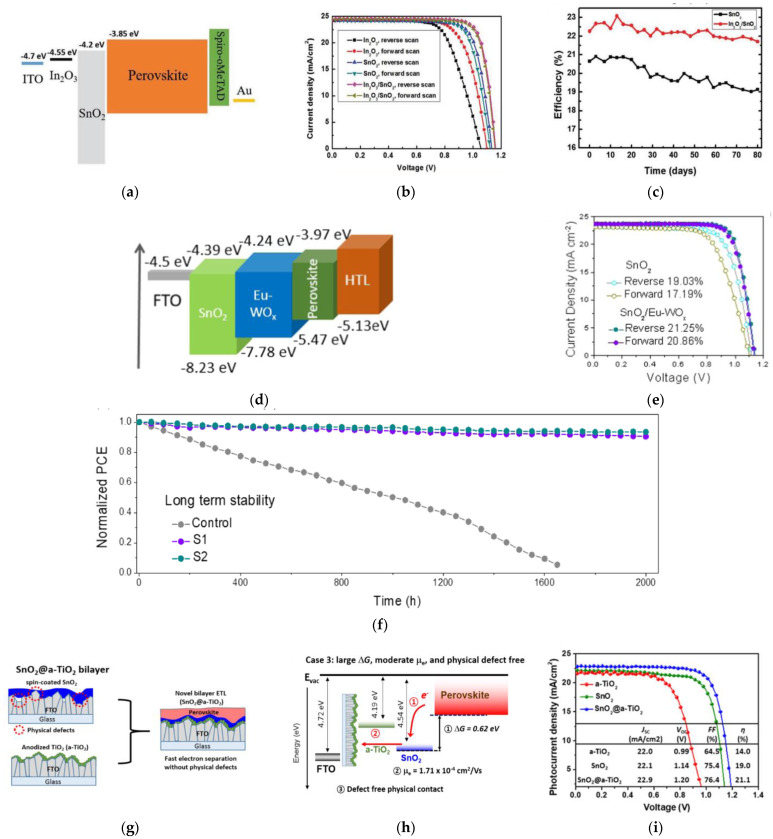
(**a**) Energy-band diagram of PSC based on In_2_O_3_/SnO_2_. (**b**) Illuminated *J*-*V* curves under 1 SUN for forward- and reverse-voltage scans of In_2_O_3_, SnO_2_, and In_2_O_3_/SnO_2_-based PSC devices. (**c**) Long-term stability measurements of devices with different ETLs. Reproduced from [31] with permission from Wiley, 2020 (**d**) Energy-band diagram of each layer in PSCs. (**e**) Illuminated *J*-*V* curves under 1 SUN under reverse- and forward-voltage scanning of PSC devices with different ETLs. (**f**) Long-term stability measurements of PSCs with different ETLs in the ambient. Reproduced from [32] with permission from Elsevier, 2021. (**g**) Schematic illustration of bilayered TiO_2_/SnO_2_ ETL. (**h**) Illustration of TiO_2_/SnO_2_ ETLs with large change in free energy (Δ*G*) and moderate electron mobility (*μ_e_*). (**i**) Illuminated *J*-*V* curves under 1 SUN for various ETLs. Reproduced from [33] with permission from ACS Publications, 2017.

Binary layers of SnO_2_ with other metal oxides is another approach to modify the ETL in PSCs. This approach leads to tailoring the surface morphology and tunes the energy band alignment. Wang et al. investigated inserting indium oxide (In_2_O_3_) between ITO and SnO_2_, which resulted in reduced trap densities in the perovskite with improved charge transfer and band alignment [31], as shown in Figure 2a. This resulted in higher *V_OC_* and *FF* values with a PCE enhancement of 21.4% to 23.1%, as shown in Figure 2b. Unsealed devices retained 98% of its initial efficiency after being stored in N_2_ for 1920 h, as shown in Figure 2c, resulting in 91% of its initial PCE after 180 h of continuous 1 SUN illumination and about 80% of its initial PCE after 120 h of exposure to 75% relative humidity.

Europium (Eu) doped tungsten-oxide (WO*_x_*) nanorods were inserted between the perovskite and SnO_2_ layer by Chen et al. [32], which contributed to improved crystallinity of the perovskite film and enhanced conductivity and carrier mobility of both the SnO_2_ ETL and spiro-OMeTAD HTL. Due to the energy level of the Eu:WO*_x_* nanorods, as shown in Figure 2d, the electron and hole extraction were remarkably boosted at the ETL/absorber and absorber/HTL interface, improving the PV performance, as shown in Figure 2e. Unencapsulated devices retained over 90% of its initial PCE after 500 h of continuous 1 SUN illumination at 16–25 °C and 20–30% relative humidity, and over 90% of its initial PCE after 2000 h of exposure to ambient air, as shown in Figure 2f.

Song et al. investigated inserting an anodized-TiO_2_ between the FTO and SnO_2_ ETL, which lead to a defect-free physical contact and improved electron extraction [33], as illustrated in Figure 2g. Such bi-layered ETLs resulted in a large change in free energy and moderate electron mobility, as illustrated in Figure 2h. This enhanced the *V_OC_* from 1.14 to 1.20 V, which led to an enhanced PCE of 19.0% to 21.1%, as shown in Figure 2i. Similarly, Lee et al. investigated combining a compact TiO_2_ between the TCO and SnO_2_ ETL, which improved charge collection due to better hole-blocking ability of the TiO_2_ underlayer [34]. This resulted in increasing the PCE from 16.4% to 19.8%.

Unencapsulated devices maintained over 95% of its initial PCE after 1200 h of storage in air with 20% relative humidity.

Dagar et al. studied inserting a thin magnesium-oxide (MgO) overlayer on top of SnO_2_, which led to more uniform films and reduced interfacial-carrier recombination [35]. This resulted in better stability and enhanced device performance from a PCE of 15.2% to 19.0%. Unsealed devices maintained 67% of its initial PCE after 2568 h in 30% relative humidity.

A bilayer of lead oxide (PbO) doped-SnO_2_ with undoped SnO_2_ was investigated by Bi et al. [36], which improved the shunt resistance and enhanced the *FF* from 72.9% to 75.5% and PCE from 17.0% to 18.8%. Devices maintained over 90% of its initial efficiency after 1080 h at room temperature with 15% relative humidity.

Ultrathin MgO was also inserted between FTO and SnO_2_ ETL in PSCs by Ma et al., which resulted in enhanced electron transporting and hole-blocking properties [37]. Due to MgO passivation, less FTO-surface defects were observed along with a smoother surface and suppressed carrier recombination. The PCE increased from 16.4% to 18.2%.

Hou et al. studied chemical-bath deposition of a SnO_2_/TiO_2_ bilayer [38], which facilitates charge separation achieving effective extraction and transport of electrons. Higher electron mobility and suppressed recombination was observed due to the reduced energy barriers and gradual-energy levels, which lead to a PCE enhancement of 12.0% to 18.1%. 

Yan et al. reported a bi-layered ETL of SnO_2_ and zinc oxide (ZnO), which showed superior electron extraction and a lower charge recombination rate [39]. This resulted in a *V_OC_* enhancement of 1.06 to 1.23 V, and a PCE enhancement of 11.9% to 14.6%. Unencapsulated devices retained 80% of its initial efficiency after 300 h in N_2_ at 85 °C.

### 2.3. Ionic Compounds

#### 2.3.1. Surface Modification by Ionic Compounds

Adding ionic compounds into the SnO_2_ solution or applying it to the surface the SnO_2_ are some other cost-effective approaches to modifying the SnO_2_ ETL. Compared to organic molecules and carbon materials, ionic compounds are usually more stable and lower in cost.

**Figure 3:**
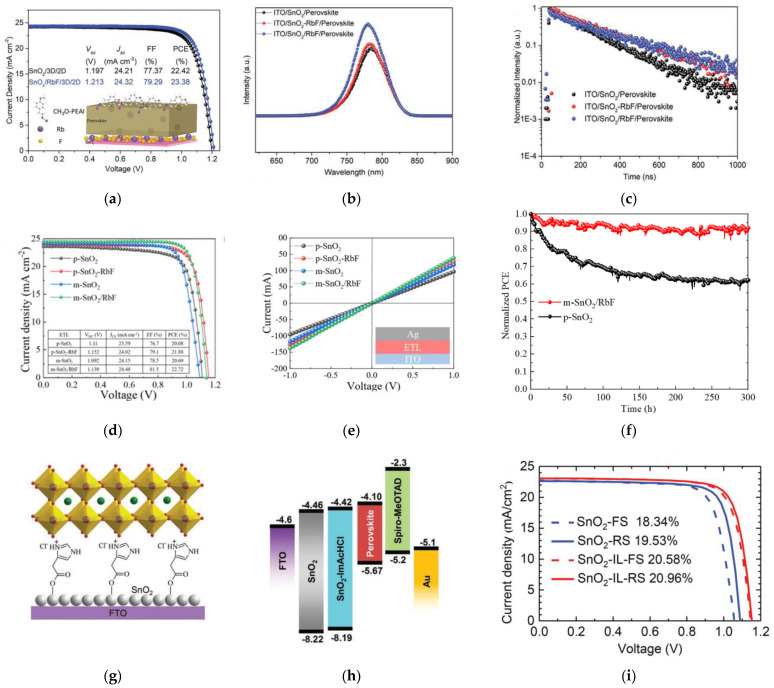
(**a**) Illuminated *J*-*V* curves under 1 SUN for forward- and reverse-voltage scanning of SnO_2_ and SnO_2_/RbF-based PSC devices. (**b**) Steady-state PL and (**c**) TRPL spectra of perovskite films on various ETLs. Reproduced from [40] with permission from Wiley, 2021 (**d**) Illuminated *J*-*V* curves under 1 SUN for PSCs based on various ETLs. (**e**) Current vs. voltage (*I*-*V*) characteristic curves for various ETLs with an ITO/ETL/Ag structure. (**f**) Long-term maximum power point tracking (MPPT) stability measurements of devices with different ETLs under continuous 1 SUN illumination (simulated by LED light). Reproduced from [41] with permission from Elsevier, 2022. (**g**) Schematic illustration of formation of ImAcHCl-modified SnO_2_ ETL. (**h**) Energy-band diagram of each layer in PSCs. (**i**) Illuminated *J*-*V* curves under 1 SUN for various ETLs. Reproduced from [42] with permission from Wiley, 2019.

Zhuang et al. looked into the modification of SnO_2_ by using rubidium fluoride (Rb) by using two different methods: (i) adding RbF into the SnO_2_ solution and (ii) inserting RbF at the SnO_2_/perovskite interface [40]. Adding RbF to the SnO_2_ bulk resulted in improved electron mobility, while adding RbF to the surface of SnO_2_ resulted in inhibited ion migration and reduced carrier recombination due to the Rb^+^ cations escaping into the bulk perovskite. This led to increased *V_OC_* with PCE of over 23%, as shown in Figure 3a with negligible hysteresis. A stronger steady-state PL intensity of the RbF-treated SnO_2_ corresponds to improved perovskite-film quality, as shown in Figure 3b. Based on the TRPL results in Figure 3c, a shorter fast decay (*τ*_1_) indicates enhanced electron extraction, while the longer slow decay (*τ*_2_) corresponds to decreased defects/traps and improved perovskite-film quality. Unencapsulated PSCs maintained about 75% of its initial PCE after 200 h of exposure to white LED light illumination.

Chen et al. studied the combined effect of doping planar-SnO_2_ (p-SnO_2_) with different concentrations of RbF and depositing RbF onto the mesoporous SnO_2_ (m-SnO_2_) layer [41], resulting in improved PV performance, as shown in Figure 3d. RbF modification increased the conductivity of SnO_2_, as shown in Figure 3e, and passivated interfacial traps through F-Sn bonds. Rb^+^ diffused into the perovskite which passivated the perovskite and suppressed ion migration. Unencapsulated devices maintained 90% of its initial PCE after 300 h at the maximum power point (MPP) under 1 SUN illumination, as shown in Figure 3f.

Chen et al. reported surface modification of the SnO_2_ by using 4-imidazoleacetic acid hydrochloride (ImAcHCl) [42], as illustrated in Figure 3g. The chloride anion in ImAcHCl improves the crystallinity of the perovskite layer. Modifying SnO_2_ with ImAcHCl shifts the conduction and valence bands up, as shown in Figure 3h, suppresses carrier recombination, and enhances carrier lifetime. This results in the enhancement of PCE from 19.5% to 21.0%, as shown in Figure 3i. Unencapsulated-PSC devices retained 95% of its initial PCE after 840 h at room temperature with 46–60% relative humidity. Unencapsulated PSCs based on PTAA maintained 90% of its initial PCE after 40 h of exposure to 85 °C in N_2_.

Potassium-hydroxide (KOH) modification of SnO_2_ surfaces was investigated by Bu et al., which resulted in suppressed hysteresis and enhanced PV performance with a PCE increase from 19.3% to 20.5% [43]. Potassium cations were shown to passivate the ETL/perovskite interface, facilitate grain growth of the perovskite, and improve stability.

Cesium-carbonate (Cs_2_CO_3_) post-treatment, studied by Li et al., improves the electrical properties of SnO_2_ and passivates the ETL/perovskite interface [44]. Such Cs_2_CO_3_ modification improves the surface wettability of the ETL and reduces the roughness, resulting in a perovskite film with larger grains. The Cs_2_CO_3_ post-treatment lowers the work function, reducing electron-hole recombination, and enhancing the electron transfer. Devices retained 91% of its initial efficiency after stored in 35–45% relative humidity for 340 h.

Alkali-metal cations, such as lithium chloride (LiCl), sodium chloride (NaCl), potassium chloride (KCl), rubidium chloride (RbCl), and cesium chloride (CsCl), were used to modify the SnO_2_ surface [45]. Such modification increases the mobility and reduces the trap density of states of SnO_2_. Efficient defect passivation suppresses the recombination at the ETL/absorber interface. Devices based on NaCl-treated SnO_2_ maintained over 90% of its initial PCE after 960 h.

#### 2.3.2. Bulk Incorporation of Ionic Compounds

**Figure 4:**
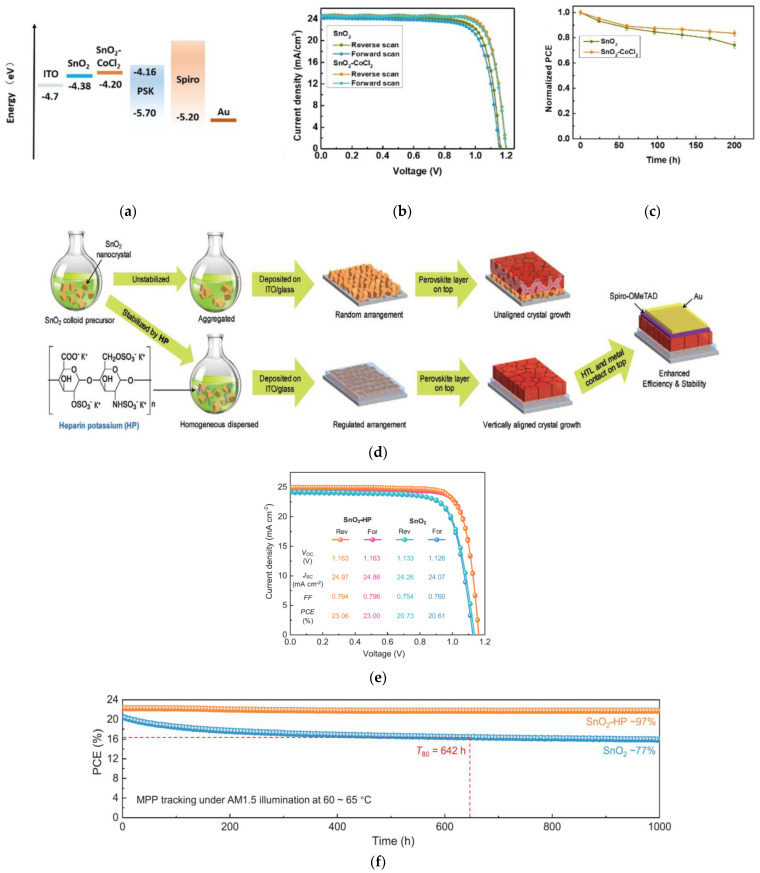

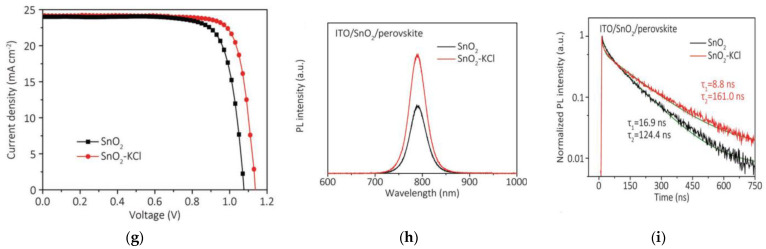
(**a**) Energy-band levels of each layer in the PSC. (**b**) Illuminated *J*-*V* curves under 1 SUN for reverse- and forward-voltage scanning of SnO_2_ and CoCl_2_:SnO_2_-based PSC devices. (**c**) Stability measurements of devices with various ETLs in N_2_ atmosphere under continuous irradiation. Reproduced from [46] with permission from ACS Publications, 2021. (**d**) Illustration of incorporation of heparin potassium (HP) into the SnO_2_ nanocrystal dispersions, resulting in arrangements of ETL nanocrystals, and crystal growth of the perovskite films with and without heparin potassium. (**e**) Illuminated *J*-*V* curves under 1 SUN for reverse- and forward-voltage scans of PSCs with and without HP incorporation into SnO_2_. (**f**) Long-term MPPT-stability measurements of devices with different ETLs for 1000 h under continuous 1 SUN illumination. Reproduced from [47] with permission from Wiley, 2020. (**g**) Illuminated *J*-*V* curves under 1 SUN for various ETLs. (**h**) Steady state PL and (**i**) TRPL spectra for various ETLs. Reproduced from [48] with permission from Wiley, 2020.

Cobalt chloride hexahydrate (CoCl_2_·6H_2_O) was incorporated into SnO_2_ by Wang et al. which shows better band alignment, as shown in Figure 4a; it enhanced charge extraction and suppressed interfacial recombination [46]. This enhanced the *V_OC_* up to 1.20 V for a perovskite layer with a bandgap of 1.54 eV. PSC devices with a PCE of 23.8% was achieved, as shown in Figure 4b, with enhanced stability maintaining 84% of initial efficiencies after 200 h of continuous light exposure, as shown in Figure 4c.

The contact between the SnO_2_ and perovskite was improved by introducing a biological polymer, heparin potassium, to the SnO_2_ [47]. Such bulk incorporation regulated the arrangement of SnO_2_ nanocrystals and induced vertically aligned crystal growth of the perovskite, as illustrated in Figure 4d. This improved the PV performance, as shown in Figure 4e. Due to the strengthened interface binding, device-operational stability was enhanced resulting in maintaining 97% of the initial efficiency after 1000 h under 1 SUN illumination at the MPP, as shown in Figure 4f.

Introducing KCl to the SnO_2_ ETL passivated both the grain boundaries of the perovskite and the defects at the ETL/absorber interface [48]. The Cl^−^ and K^+^ ions passivate the ETL/absorber contact, while the K^+^ ions in the ETL diffuse into the perovskite layer and passivate the grain boundaries, resulting in enhanced *V_OC_* from 1.08 to 1.14 V and increased PCE from 20.2% to 22.2%, as shown in Figure 4g. The stronger steady-state PL intensity with the presence of KCl demonstrates suppressed recombination of the perovskite, as shown in Figure 4h. Based on TRPL results in Figure 4i, the decrease in *τ*_1_ suggests faster electron transfer with the incorporation of KCl, and the increase in *τ*_2_ suggests slower recombination in the perovskite film grown on KCl-incorporated SnO_2_. Unsealed devices maintained 88% of its initial PCE after 120 h of continuous 1 SUN illumination.

Girard’s Reagent T (GRT) was introduced to the SnO_2_ nanoparticles by Bi et al. [49]. The carbonyl group in GRT is anticipated to prevent agglomeration of the SnO_2_ nanoparticles. The quaternary-ammonium-chloride salt in GRT is expected to facilitate the crystal growth of the perovskite, and the quaternary-ammonium cation and chloride anion can passivate the defects at the ETL/absorber interface. Such GRT incorporation into SnO_2_ resulted in better electrical properties of SnO_2_, promoted vertical growth of the perovskite, and reduced interfacial defects at the ETL/perovskite interface, resulting in a PCE enhancement of 19.8% to 21.6%. Unencapsulated devices retained over 99% of its initial efficiency after 720 h at 60 °C, and 59% after 672 h under 1 SUN illumination. 

Liu et al. introduced ammonium chloride (NH_4_Cl) into SnO_2_, which resulted in PSC devices with negligible hysteresis and improvement in PCE from 18.7% to 21.4% [50]. Such improvement is due to the increased electron mobility, and improved band alignment and passivation of the ETL/perovskite interface, which also improved the device’s stability. Unencapsulated devices stored in N_2_ retained over 95% after 1000 h.

Sun et al. incorporated potassium sodium tartrate (PSTA) into the SnO_2_ colloidal dispersion [51]. PTSA contains mobile-alkali-metal cations leading to improved uniformity and conductivity, and less defects in SnO_2_, which improves the crystallinity of the perovskite film. Sodium and potassium cations can diffuse into the perovskite and passivate the defects at the grain boundaries and surface. This results in PCE enhancement of 18.3% to 21.1% with reduced hysteresis. Device stability improves with unencapsulated devices retaining over 95% of its initial efficiency after 1440 h of exposure to ambient air with 45% relative humidity at 25 °C.

Phosphoric acid was introduced into SnO_2_ by Jiang et al. to eliminate dangling bonds on the SnO_2_ surface and improve the carrier-collection efficiency [52]. Electron mobility increased by 3 times and surface-trap states reduced, decreasing the electron- transport barriers of SnO_2_. Attributed to the enhanced electron-collection efficacy, the PCE increased from 19.7% to 21.0%.

Tetramethylammonium hydroxide (TMAH) was incorporated into SnO_2_ by Huang et al. at low temperatures of 100–150 °C [53]. Such modification of SnO_2_ attributed to higher conductivity of SnO_2_ and also effectively passivated the grain boundaries of the perovskite film. Improved charge transport between the perovskite and ETL resulted in enhanced efficiencies from 18.1% to 20.5%. Encapsulated devices in 15% relative humidity maintained 97% of its initial PCE after 360 h.

Modification of SnO_2_ by introducing cesium fluoride (CsF) into the ETL was investigated by Akin et al., which led to improved optoelectronic properties and rapid extraction of photogenerated electrons [54]. By combining the modification of SnO_2_ and inserting zwitterion molecules at the perovskite/HTL interface, a high *V_OC_* value of 1.23 V and a PCE value of 20.5% were achieved. Device-operational stability was also improved retaining over 90% of its initial PCE after 800 h under continuous 1 SUN illumination at the MPP.

Ammonium sulfide [(NH_4_)_2_S] is incorporated in SnO_2_ reported by Ai et al., which passivated the surface defects by terminating the Sn-S dangling bonds [55]. The conductivity and electron mobility of SnO_2_ are increased, enhancing the electron collection and lowering electron-hole recombination rate. The Sn-S-Pb anchors the perovskite crystals at the ETL/absorber interface, which enhances the stability and electron extraction of the PSC. PCE values increase from 18.7% to 20.0% by using this method.

### 2.4. Carbon Materials

#### 2.4.1. Surface Modification by Carbon Materials

**Figure 5:**
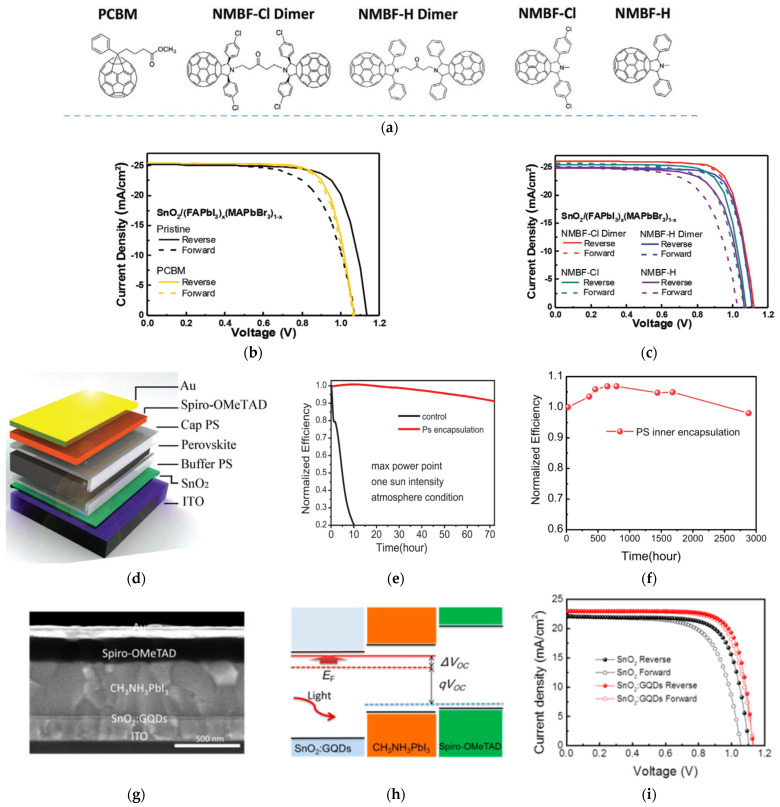
(**a**) Chemical structures of fullerene dimers and monomers. Illuminated *J*-*V* curves under 1 SUN for reverse- and forward-voltage scans of (**b**) SnO_2_ and SnO_2_/PCBM-based, and (**c**) SnO_2_/NMBF-Cl and SnO_2_/NMBF-H monomer and dimer-based PSC devices. Reproduced from [56] with permission from Wiley, 2020. (**d**) Schematic of PSC-device stack based on polystyrene (PS) modified SnO_2_ ETL. (**e**) Stability tests at the MPP under 1 SUN illumination. (**f**) Long-term stability measurements of devices with inner-PS encapsulation stored in air. Reproduced from [57] with permission from Wiley, 2019. (**g**) Cross-sectional scanning electron microscopy (SEM) image of device with SnO_2_/GQDs ETL. (**h**) Energy-band levels of device based on SnO_2_/GQDs before and after illumination. (**i**) Illuminated *J*-*V* curves under 1 SUN for various ETLs. Reproduced from [58] with permission from ACS Publishing, 2017.

Insertion of highly conductive carbon material at the ETL/absorber interface can facilitate electron transfer. A smooth ETL surface can also be enabled to impact the growth and nucleation of the perovskite layer on top. In addition, defects at the ETL/absorber interface can be passivated by the carbon material. For example, Wang et al. investigated the application of novel fulleropyrrolidine (NMBF-X, X = H or Cl) monomers and dimers, as shown in Figure 5a, in between the perovskite and ETL [56]. The chlorinated- fullerene dimers resulted in the most efficient PCE of 22.3% with minimal hysteresis, as shown in Figure 5b,c, which stems from the passivation of the NMBF-Cl dimer with the SnO_2_ and perovskite simultaneously. After 1000 h, unencapsulated PSC devices exposed to air at 25–35 °C with 45–60% relative humidity retained over 95% of its initial PCE.

Polystyrene (PS) was inserted between the ETL and absorber layer to release residual stress in the absorber during annealing, which attributes to reduced interface defects, less recombination, and lower ion-migration tendencies [57]. PS was also applied on top of the perovskite film for inner-encapsulation, as shown in Figure 5d, which improves the long-term device stability maintaining over 90% or its initial efficiency after 72 h at the MPP under continuous 1 SUN illumination, as shown in Figure 5e. Devices with the PS- inner encapsulation maintained over 90% of its initial efficiency after 2800 h in air, as shown in Figure 5f.

Adding graphene quantum dots (GQDs) to the SnO_2_ surface, as shown in Figure 5g, will improve the conductivity and fill the electron traps in SnO_2_, which improves the electron extraction and reduces the recombination at the ETL/absorber interface [58]. PSC devices with GQD-treated SnO_2_ results in a *V_OC_* enhancement of 1.10 to 1.13 V and a PCE enhancement of 17.9% to 20.3% with little hysteresis, as shown in Figure 5h,i. Unencapsulated devices maintain over 95% of its initial efficiency in N_2_ after 720 h and then an additional 1440 h in air (20–30% relative humidity).

Zheng et al. reported modifying the SnO_2_ ETL surface with [6,6]-phenyl-C_61_-butyric acid methyl ester (PCBM) [59]. By simultaneously inserting PbS above the HTL and spiro-OMeTAD, a PCE of 19.6% was demonstrated with long-term storage stabilities of about 100% retained after exposure to air for 1000 h.

Ke et al. also reported fullerene, PCBM, and modification of the SnO_2_ ETL surface, which resulted in an increase in PCE from 16.5% to 19.1% [60]. PCBM promotes enhanced electron transfer, suppressed carrier recombination at the ETL/absorber interface, and passivates both the ETL/absorber interface and the perovskite-grain boundaries.

Plasma-enhanced atomic layer deposition (PEALD) was reported to use a low deposition temperature of 100 °C and still attained high efficiencies by surface modification with passivation by C_60_-self-assembled monolayer (C_60_-SAM) [61]. The *V_OC_* improved from 1.07 to 1.13 V and *FF* increased from 75.5% to 79.1%, resulting in an efficiency enhancement from 17.2% to 19.0%. Unencapsulated devices maintained over 98% of its initial PCE after 480 h in N_2_ with room-light exposure and a relative humidity below 10%.

A comparative study between passivation of SnO_2_ by [6,6]-phenyl-C_61_-butyric acid (PCBA) and PCBM was performed by Wang et al. [62]. Passivation of fullerene-molecules at the SnO_2_ surface efficiently decreases the defects and improves the conductivity through enhanced electron mobility. The PCBA modification showed higher passivation efficiency, resulting in a PCE increase from 15.4% to 18.6%. 

#### 2.4.2. Bulk Incorporation of Carbon Materials

**Figure 6:**
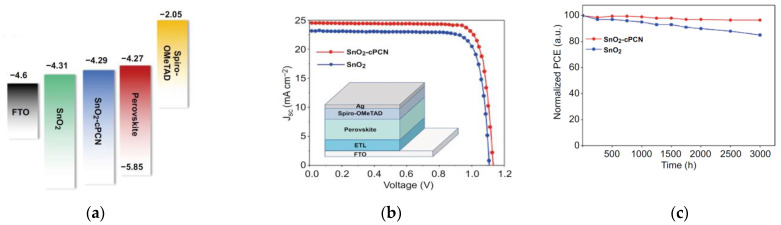

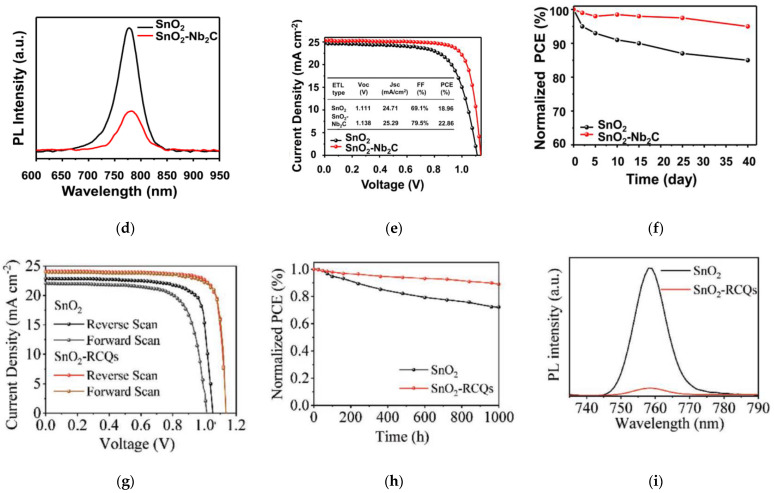
(**a**) Energy-band level of each layer in the PSC. (**b**) Illuminated *J*-*V* curves under 1 SUN for SnO_2_ and cPCN:SnO_2_-based devices. (**c**) Long-term stability measurements of unencapsulated devices in N_2_. Reproduced from [63] with permission from Springer, 2021. (**d**) PL spectra of perovskite film grown on various ETLs. (**e**) Illuminated *J*-*V* curves under 1 SUN for SnO_2_ and Nb_2_C:SnO_2_-based devices. (**f**) Long-term stability measurements of devices with different ETLs. Reproduced from [64] with permission from Elsevier, 2021. (**g**) Illuminated *J*-*V* curves under 1 SUN for reverse and forward scans of SnO_2_ and carbon quantum dot:SnO_2_-based devices. (**h**) Long-term stability measurements of unencapsulated devices with different ETLs in ambient dark environment at 25 °C with 40–60% relative humidity. (**i**) Steady-state PL spectra of perovskite on varying ETLs. Reproduced from [65] with permission from Wiley, 2020.

Highly conductive carbon-based materials incorporated into the SnO_2_ can enhance the conductivity of the ETL. For example, polymeric carbon nitrides (cPCNs) were introduced into the SnO_2_ nanocrystals by Li et al., which led to electron mobilities three times higher than that of the undoped SnO_2_ [63]. Such modification of SnO_2_ led to less wettability, reduced grain boundaries from the suppressed heterogeneous nucleation of the perovskite, and improved the band alignment, as shown in Figure 6a. This resulted in negligible hysteresis with a PCE enhancement of 21.3% to 23.2%, as shown in Figure 6b. Unencapsulated devices retained 95% of its initial PCE after 2880 h in N_2_, as shown in Figure 6c.

Niu et al. studied the effect of incorporating Nb_2_C MXenes into SnO_2_, which led to increased grain growth and increased lattice-spacing facets of SnO_2_ [64]. A decrease in the steady-state PL, shown in Figure 6d, demonstrates enhanced photogenerated carrier generation and reduced recombination. Superior crystallinity and effective carrier transport resulted in improved PSC PCE from 19.0% to 22.9%, as shown in Figure 6e. Unencapsulated devices retained 98% of its initial PCE after 960 h in 40–60% relative humidity at 25 °C, as shown in Figure 6f.

Red-carbon quantum dots were used to dope solution-processed SnO_2_, which resulted in increasing the electron mobility by 20 times [65]. The enhanced electron mobility also showed to help passivate the traps and defects at the ETL/perovskite interface and promote growth of highly crystalline perovskite. Efficiencies improved from 19.2% to 22.8% through improvement of *J_SC_*, *V_OC_*, and *FF*, as shown in Figure 6g. Unencapsulated devices maintained 96% of its initial PCE after 1000 h in air with 40–60% relative humidity at 25 °C, as shown in Figure 6h. Reduced steady-state PL intensity of the perovskites grown on the SnO_2_ doped with red-carbon quantum dots suggest improved photogenerated-carrier generation and suppressed recombination, as shown in Figure 6i.

A water-soluble nonionic polymer, polyacrylamide (PAM), is introduced into SnO_2_ be Dong et al., which improves the electron mobility, wettability, and uniformity of SnO_2_ [66]. Defects in the perovskite is also reduced and grain size is increased from the PAM addition into SnO_2_. Band alignment at the ETL/perovskite interface is also improved, resulting in a PCE enhancement from 20.2% to 22.6%. Unencapsulated devices maintained 90% of its initial efficiency after 1080 h of exposure to 45%–55% humidity.

Graphitic carbon nitride (g-C_3_N_4_) quantum dots were added to SnO_2_ and applied to PSC devices by Chen et al. [67]. The oxygen-vacancy-reduced trap centers were effectively eliminated and bulk and interface-electron transport were promoted. The high conductivity and suitable energy band alignment of g-C_3_N_4_-treated SnO_2_ led to a PCE improvement of 20.2% to 22.1%. Unencapsulated-PSC devices retained 90% of its initial efficiency after 1500 h in air with 60% relative humidity at 25 °C, and 80% of its initial efficiency after 75 h in air with 60% relative humidity at 85 °C.

Zhang et al. incorporated graphdiyne into SnO_2_, which led to improved electron mobility [68]. The enhanced hydrophobicity inhibits heterogeneous-perovskite nucleation, attributing to the reduced grain boundaries and less defect density. The ETL/absorber interface is also improved from passivation of the Pb-I anti-site defects. This results in a PCE increase of 19.2% to 21.1%.

Zhao et al. introduced naphthalene-diimide graphene into nanocrystal SnO_2_ [69]. Such modification increases the surface hydrophobicity of SnO_2_ and forms a van-der-Waals interaction at the ETL/perovskite interface. Enhanced *FF* values from 74.6% to 82.1% are attributed to the enhanced electron mobility and electron-extraction efficiency, and reduced carrier recombination. PCE improves from 19.0% to 20.2%.

Carbon nanodots by a hydrothermal process were incorporated into SnO_2_ by Wang et al. using a simple solution process [70]. Incorporation of carbon nanodots into SnO_2_ reduced the density of trap-states and increased the electron mobility of SnO_2_, resulting in a PCE of 20.0% with negligible hysteresis. As carbon materials exhibit high conductivity, incorporation of a carbon material into the ETL can enhance its charge transport and conductivity. Carbon materials can also passivate defects, improving the stability and efficiency of PSCs. Unencapsulated PSCs maintained 90% of its initial efficiency after 200 h of UV exposure in air with 20–30% relative humidity at 20 °C.

### 2.5. Organic Molecules

#### 2.5.1. Surface Modification by Organic Molecules

**Figure 7:**
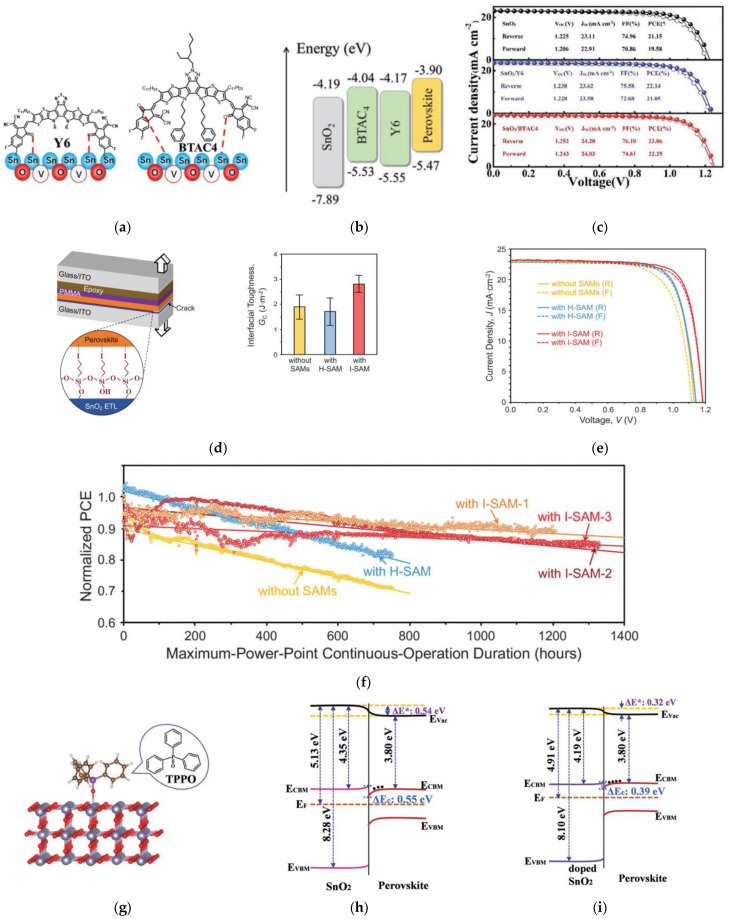
(**a**) Illustration of mechanism of oxygen-vacancy-defect passivation of Y6 and BTAC4. (**b**) Energy-level diagrams of each layer in the PSC. (**c**) Illuminated *J*-*V* curves under 1 SUN for reverse and forward scans of SnO_2_, SnO_2_/Y6, and SnO_2_/BTAC4-based devices. Reproduced from [71] with permission from Wiley, 2021. (**d**) Schematic of toughness testing and toughness results with various ETLs. Inset shows illustration of idealized I-SAM. (**e**) Illuminated *J*-*V* curves under 1 SUN for reverse and forward scans of SnO_2_, SnO_2_/H-SAM, and SnO_2_/I-SAM based devices. (**f**) Long-term stability measurements of unencapsulated devices with various ETLs at the MPP in N_2_ atmosphere under continuous 1 SUN illumination. Reproduced from [72] with permission from American Association for the Advancement of Science (AAAS), 2021. (**g**) The relaxed model of a TPPO molecule absorbed on SnO_2_ (110) surface. Energy-level band diagrams of ETL/perovskite heterojunction (**h**) without and (**i**) with TPPO-surface treatment of SnO_2_. Reproduced from [73] with permission from Wiley, 2019.

Modification of SnO_2_ by organic molecules can passivate defects at the ETL surface and improve the electrical properties and transport at the interface. The ETL and absorber layer can be chemically bridged by the organic molecule enhancing the interfacial-electron transfer. For example, Lou et al. investigated the introduction of π-conjugated *n*-type small organic molecules (BTAC4 and Y6) onto the surface of KCl-doped SnO_2_ ETL [71], as illustrated in Figure 7a, which yields less trap states, suppressed carrier recombination, and improved electron transport and extraction. Band alignment improves with the modification, as shown in Figure 7b. Applying BTAC4 and Y6 to the surface of SnO_2_ results in an enhanced PCE from 21.2% to 23.1% and 22.1%, respectively, as shown in Figure 7c. Unencapsulated devices maintained about 90% of its initial efficiency after 768 h in air with 35% relative humidity.

An iodine-terminated SAM, 3-iodopropyl trimethoxysilane [Si(OCH_3_)_3_(CH_2_)_3_I, I-SAM] was applied to the SnO_2_ ETL surface in PSCs, which increased the adhesion toughness at the ETL/absorber interface-enhancing mechanical reliability [72], as shown in Figure 7d. This is attributed to the higher toughness and decreased hydroxyl groups at the interface. Without the SAMs treatment, irreversible morphological degradation, such as voids and delamination, was observed at the ETL/perovskite interface for operational stability tested-devices. Treatment with I-SAM on the SnO_2_ enhanced the PCE from 20.2% to 21.4% with diminished hysteresis, as shown in Figure 7e. Long-term working stability was also improved, retaining over 90% of its initial efficiency in N_2_ with continuous 1 SUN illumination at the MPP for unencapsulated devices for 1200 h, as shown in Figure 7f.

Triphenylphosphine oxide (TPPO) is an air-robust and cost-effective molecule for *n*-type doping of SnO_2_ [73], as shown in Figure 7g. Surface modification by TPPO enhanced the conductivity and lowered the work function of SnO_2_, as shown in Figure 7h,i. The *V_OC_* improved from 1.08 to 1.11 V and PCE improved from 19.0% to 20.7% attributed to the lower recombination rate and faster electron extraction.

Thiophene-based interlayers were adopted to the SnO_2_ surface to reduce the energy loss by optimizing the surface-electronic states of SnO_2_ and improving the perovskite- film quality [74]. Surface modification of SnO_2_ by thiophene-3-acetic acid improved the conductivity and lowered the work function of SnO_2_. Ion-defect states at the ETL/perovskite interfaces were passivated by bonding of the under-coordinated Pb^2+^ of MAPbI_3_ with the sulfur atoms of the thiophene rings with a lone pair of electrons. *V_OC_* improved from 1.07 to 1.12 V, and *FF* improved from 73.5% to 80.1%, resulting in a PCE improvement of 17.5% to 20.6%. This resulted in improved device stability retaining over 90% or its initial efficiency after 1440 h in N_2_ and over 80% of its initial efficiency after 130 h in air with 70% relative humidity at 85 °C.

Aminosulfonic acid (^+^H_3_N-SO_3_^−^, SA) is introduced to the surface of SnO_2_ [75], and a chemical bridge is formed between the ETL and perovskite through the coordination bond to SnO_2_ via -SO_3_^−^ anions and electrostatic interactions with the perovskite via -NH_3_^+^ cations. Better surface wettability of the SA-treated SnO_2_ led to larger grain size of perovskite films. Attributed to the passivated-contact defects, *V_OC_* improved from 1.11 to 1.15 V, while barrier-free charge transferred led to improved *FF* and *J_SC_* with reduced hysteresis, resulting in a PCE improvement of 18.2% to 20.4%. Unencapsulated devices maintained over 80% of its initial PCE after 1000 h in air of 25–35% relative humidity and over 75% of its initial PCE after 500 h in N_2_ at 60 °C.

The organic molecule p-amino benzenesulfonic acid (ABSA) was introduced to the surface of SnO_2_ by inactivating the under-coordinated Sn ions [76]. This decreased the energy-band barrier on the surface of SnO_2_, and increased the conductivity and lessens- carrier recombination. This results in a PCE enhancement of 18.0% to 20.3%. Unsealed PSC devices retain 57% of its initial efficiency after 720 h in N_2_.

A plant-photosynthesis promoter, choline chloride, was introduced to the surface of SnO_2_ by a simple molecular self-assembly method [77]. Such modification reduces the oxygen vacancies of SnO_2_, while the Cl ions form strong Pb-Cl bonds with the uncoordinated Pb ions in the MAPbI_3_. This passivates the defects at the ETL/absorber interface and reduces carrier recombination, improving the *V_OC_* from 1.07 to 1.15 V. PCE is improved from 16.8% to 18.9%.

Zuo et al. investigated various SAMs, such as 4-pyridinescarboxylic acid (PA-SAM), 4-cyanobenzoic acid (CBA-SAM), and benzoic acid (BA-SAM), and applied them to the surface of SnO_2_ [78]. Proper interfacial interactions were shown to decrease trap- state density and enhance interfacial-charge transfer. Among the various SAMs, application of PA-SAM resulted in the highest efficiency enhancement of 17.2% to 18.8%, with a *V_OC_* enhancement from 1.06 to 1.10 V. This is due to improved electronic coupling and suppressed interfacial traps. Charge transfer at the ETL/absorber interface improved from the lowered work function.

Wang et al. reported interfacial-sulfur functionalization anchoring of SnO_2_ by using potassium hexylxanthate to modify the surface of SnO_2_ [79]. This approach effectively passivated the charge traps and suppressed carrier recombination at the interface by sulfur functionalization of the SnO_2_ surface. Functionalized-sulfur atoms can also coordinate with the under-coordinated Pb^2+^ ions at the interface. Such strategy resulted in improved device efficiencies of 16.6% to 18.4%. Unsealed devices maintained about 90% of its initial PCE after 1680 h at room temperature.

#### 2.5.2. Bulk Incorporation of Organic Molecules

**Figure 8:**
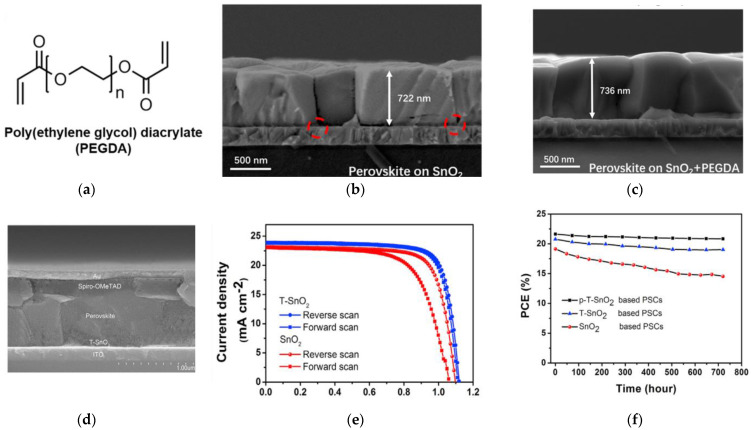

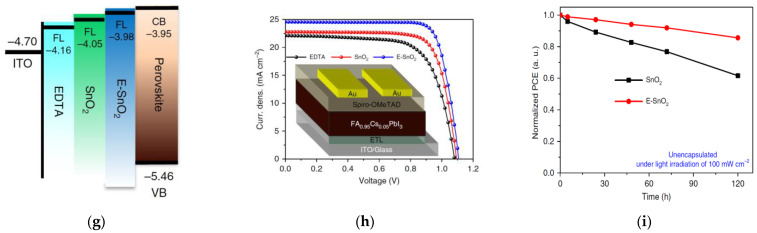
(**a**) Molecular structure of PEGDA. Cross-sectional SEM images of the perovskite on (**b**) undoped SnO_2_ and (**c**) SnO_2_ doped with PEGDA. Reproduced from [80] with permission from ACS Publications, 2021. (**d**) Cross-sectional SEM image of PSC device with SnO_2_ doped with 2,2,2-trifluoroethanol (T-SnO_2_). (**e**) Illuminated *J*-*V* curves under 1 SUN for forward and reverse scans of SnO_2_ and T-SnO_2_ based devices. (**f**) Long-term stability measurements of unencapsulated devices in air with 30–40% relative humidity for SnO_2_, T-SnO_2_, and oxygen plasma-treated T-SnO_2_ (p-T-SnO_2_) as ETLs. Reproduced from [81] with permission from Cell Press, 2019. (**g**) Fermi levels of EDTA, SnO_2_, and EDTA:SnO_2_ relative to the conduction band of the perovskite layer. (**h**) Illuminated *J*-*V* curves of PSC devices with various ETLs. (**i**) Long-term stability measurements of unencapsulated devices with different ETLs under 1 SUN illumination. Reproduced from [82] with permission from Springer Nature, 2018.

Incorporation of organic molecules possessing versatile functional groups into SnO_2_ improves the dispersion of colloids, enhances the electrical properties, and passivates the defects in SnO_2_. For example, Xiong et al. introduced poly(ethylene glycol) diacrylate (PEGDA), as shown in Figure 8a, into the SnO_2_ dispersion to prevent aggregations [80], which resulted in more uniform film and well-matched band-energy alignment with the perovskite. PEGDA-modified SnO_2_ also attributed to passivating the defects at the ETL/absorber interface, as shown in Figure 8b,c. This showed a PCE improvement from 21.8% to 23.3%, with a *V_OC_* improvement of 1.09 to 1.14 V. Unencapsulated-PSC devices maintained over 90% of its initial PCE after 850 h in N_2_ under 1 SUN illumination and 98% of its initial efficiency after 1000 h in air with 30–35% humidity. 

Luan et al. incorporated 2,2,2-trifluoroethanol (TFE) into the SnO_2_ ETL [81], as shown in Figure 8d, which showed enhanced electron mobility and optimized energy- band alignment. The modified SnO_2_ exhibits a very smooth surface, which attributed to the less trap density at the ETL/absorber interface and inside the perovskite film, leading to lessened carrier recombination. With the addition of oxygen-plasma treatments PCE values of 21.7% were achieved with *FF* over 80%, as shown in Figure 8e. Unencapsulated devices maintained over 90% of its initial efficiency after 720 h in 30–40% relative humidity, as shown in Figure 8f.

Ethylene diamine tetraacetic acid (EDTA) was incorporated into the SnO_2_ ETL by Yang et al. [82]. Electron transfer is facilitated due to the optimized energy-band alignment, as shown in Figure 8g, and enhanced electron mobility of the modified SnO_2_. Perovskite film grown on the modified SnO_2_ also exhibited larger grain size and lower trap density. This led to a PCE enhancement of 18.9% to 21.6%, as shown in Figure 8h. Unencapsulated devices maintained 86% of its initial efficiency after 120 h in continuous 1 SUN illumination, as shown in Figure 8i. The large grain size of the perovskite repressed perovskite degradation at the grain boundaries.

Polyethylene glycol (PEG) introduced into the SnO_2_ prevented nanoparticle agglomeration, resulting in a dense and uniform film [83]. Such modification of SnO_2_ improved the wettability and enabled pinhole-free perovskite films, demonstrating a PCE enhancement of 19.2% to 20.8%. Unsealed devices retained over 97% of its initial efficiency after 2160 h in air with 30–80% relative humidity at 28–35 °C.

Polyethylenimine (PEIE) was added into SnO_2_ by a low-temperature solution process [84]. Such doping of SnO_2_ resulted in optimized band alignment, larger built-in potential, improved electron transport and extraction, and mitigated charge recombination. Unencapsulated devices retained 82% of its initial efficiency after1,680 h in 40% relative humidity.

## 3. Conclusions and Future Directions

In summary, recent progress in modifying the SnO_2_-ETL bulk and surface properties are discussed. SnO_2_ has been considered the most promising alternative to TiO_2_, as it has a high electron mobility and conductivity. SnO_2_ also possesses a suitable band structure owning a deep conduction band allowing enhanced electron extraction at the ETL/absorber interface. The wide bandgap of SnO_2_ allows most of the light to be absorbed by the perovskite-absorber layer and suppresses UV-related photochemical reaction, improving the device stability. SnO_2_ can also be processed at a lower temperature than TiO_2_ allowing for flexible applications to be possible. Despite the many advantages of SnO_2_, there are drawbacks, such as defects in the surface and bulk of the pristine films, deteriorating its electronic properties. Such drawbacks can be alleviated by the various surface-modification and bulk-incorporation methods discussed in this review. 

Surface-modification and bulk-incorporation methods are discussed as strategies, including elemental doping, metal-oxides bilayers, incorporation of ionic compounds, carbon materials, and organic molecules. Among the five main aspects of modifying the ETL, bulk and surface modification using ionic compounds are generally lower in cost and more stable than carbon materials and organic molecules. Bilayer-metal-oxide approaches are simple and straightforward; however, an additional fabrication step and additional material cost is required. Generally, surface-modification approaches require an additional fabrication step, so bulk incorporation may be considered more fabrication-friendly. Various modification at the surface or the bulk can lead to improved morphology, conductivity, and band alignment of SnO_2_, resulting in improved electron-transport capabilities and reduced carrier recombination. Modification of the surface properties of SnO_2_ can also lead to improved quality and crystallinity of the perovskite absorber and reduced interfacial defects at the ETL/absorber interface, attributing to improved PV device performance and stability.

Although such modification approaches of SnO_2_ have demonstrated enhanced device performance and stability, there still requires improvement in the operational stability of the PSCs at the MPP under continuous 1 SUN illumination. There have been many reports on the long-term stability of devices exposed to elevated temperatures and high relative humidity conditions. However, there are a few reports on the MPP tracking under continuous 1 SUN illumination which are working conditions of the PSCs, and is an important evaluation of the long-term operational stability of PSCs. Such long-term working-stability evaluation methods will help create a better understanding of the mechanisms for improved working stability and will be critical for future directions for commercialization. Another important factor to consider for future directions will be the scalability of the modification approaches for the SnO_2_ ETL, since developing methods compatible with large-area substrates will be essential for commercialization. 

Perovskite-tandem applications, with lower bandgap absorbers, such as silicon, Cu(In,Ga)Se_2_, and tin-related absorbers [85,86,87,88,89,90,91], below wider bandgap perovskite-based solar cells, are also future steps to commercialization [92]. Thus, depending on the bottom- solar cell, there may be limitations in fabrication methods or temperature process of the ETL in the tandem configurations. Especially, tandem devices with flexible substrates will have a limitation on the process temperature of the layers in the top solar cell.

## Figures and Tables

**Table 1:** Summary of perovskite-solar cells based on elemental doping of SnO_2_ ETL.

ETL	Method	Device Stack	*J_SC_* (mA/cm^2^)	*V_OC_* (V)	*FF* (%)	*η* (%)	Institute, Year [Ref.]
Ta:SnO_2_	Chemical Bath Deposition	ITO/ETL/MAPbI_3_/Spiro-OMeTAD/Au	21.7 → 22.8	1.16 → 1.16	77.7 → 78.6	19.5 → 20.8	Fudan, 2019 [22]
Bilayer F:SnO_2_	Spin Coating	FTO/ETL/(FAPbI_3_)_0.85_(MAPbBr_3_)_0.15_/Spiro-OMeTAD/Au	21.7 → 22.9	1.03 → 1.13	72.3 → 78.1	16.3 → 20.2	Huazhong UST, 2018 [23]
Li:SnO_2_	Spin Coating	ITO/ETL/MAPbI_3_/Spiro-OMeTAD/Au	22.0 → 23.3	1.08 → 1.11	64.2 → 70.7	15.3 → 18.2	KIST, 2016 [24]
Nb:SnO_2_	Spin Coating	FTO/ETL/(FAPbI_3_)_0.85_(MAPbBr_3_)_0.15_/Spiro-OMeTAD/Au	21.7 → 22.4	1.06 → 1.08	65.9 → 72.7	15.1 → 17.6	Shaanxi Normal U., 2017 [25]
Y:SnO_2_	Hydrothermal Growth	FTO/ETL/MAPbI_3_/Spiro-OMeTAD/Au	19.3 → 22.6	1.05 → 1.08	66.0 → 71.0	13.4 → 17.3	Wuhan U. & Toledo, 2017 [26]
Sb:SnO_2_	Spin Coating	ITO/ETL/MAPbI_3_/Spiro-OMeTAD/Au	22.3 → 22.6	1.01 → 1.06	69.6 → 72.0	15.7 → 17.2	UNL, 2016 [27]
Ga:SnO_2_	Spin Coating	AZO/ETL/CsFAMAPb(Br,I)_3_/Spiro-OMeTAD/Au	22.0 → 22.8	1.00 → 1.07	57.0 → 70.0	12.5 → 17.0	Adolphe Merkle Inst. & HZB, 2018 [28]
Mg:SnO_2_	Spin Coating	FTO/ETL/MAPbI_3_/Spiro-OMeTAD/Au	17.4 → 21.4	0.94 → 1.00	50.0 → 70.8	8.2 → 15.2	Wuhan U., 2016 [29]
Al:SnO_2_	Spin Coating	FTO/ETL/MAPbI_3_/Spiro-OMeTAD/Au	16.8 → 19.4	1.00 → 1.03	53.0 → 58.0	9.0 → 12.1	UESTC, 2017 [30]

**Table 2:** Summary of perovskite-solar cells based on metal oxide modified SnO_2_ ETL.

ETL	Method	Device Stack	*J_SC_* (mA/cm^2^)	*V_OC_* (V)	*FF* (%)	*η* (%)	Institute, Year [Ref.]
In_2_O_3_/SnO_2_	Spin Coating	ITO/ETL/MAFAPbICl/spiro-OMeTAD/Au	24.3 → 24.5	1.13 → 1.16	78.1 → 81.2	21.4 → 23.1	Nankai, 2020 [31]
SnO_2_/Eu:WO*_x_*	Spin Coating	FTO/ETL/CsFAMAPbIBr/spiro-OMeTAD/Eu:WO*_x_*/Au	23.3 → 24.0	1.11 → 1.16	73.4 → 79.4	19.0 → 22.1	Jilin, 2021 [32]
TiO_2_/SnO_2_	Potentiostatic Anodization, Spin Coating	FTO/ETL/MAFAPbBrI/spiro-OMeTAD/Ag	22.1 → 22.9	1.14 → 1.20	75.4 → 76.4	19.0 → 21.1	Toronto, POSTECH, 2017 [33]
TiO_2_/SnO_2_	Spray Pyrolysis, Spin Coating	FTO/ETL/MAPbI_3_/PTAA/Au	21.5 → 22.6	1.10 → 1.13	69.0 → 78.0	16.4 → 19.8	EPFL, 2017 [34]
SnO_2_/MgO	Spin Coating	ITO/ETL/MAPbI_3_/spiro-OMeTAD/Au	21.3 → 22.1	1.10 → 1.13	64.9 → 75.7	15.2 → 19.0	RTV, 2018 [35]
PbO:SnO_2/_ SnO_2_	Spin Coating	FTO/ETL/MAPbI_3_/spiro-OMeTAD/Au	20.9 → 22.6	1.11 → 1.10	72.9 → 75.5	17.0 → 18.8	CAS, 2021 [36]
MgO/SnO_2_	Spin Coating	FTO/ETL/MAPbI_3_/spiro-OMeTAD/Au	21.6 → 22.7	1.07 → 1.10	71.0 → 73.0	16.4 → 18.2	Wuhan, 2017 [37]
SnO_2_/TiO_2_	Chemical Bath Deposition	FTO/ETL/MAPbI_3_/spiro-OMeTAD/Ag	22.2 → 22.5	0.97 → 1.01	56.0 → 79.0	12.0 → 18.1	ECUST, Griffith, 2017 [38]
SnO_2_/ZnO	Spin Coating	ITO/ETL/CsPbI_2_Br/spiro-OMeTAD/MoO_3_/Ag	14.7 → 15.0	1.06 → 1.23	75.7 → 78.8	11.9 → 14.6	South China UT, 2018 [39]

**Table 3:** Summary of perovskite-solar cells based on surface modification of SnO_2_ ETL by ionic compounds.

ETL	Method	Device Stack	*J_SC_* (mA/cm^2^)	*V_OC_* (V)	*FF* (%)	*η* (%)	Institute, Year [Ref.]
SnO_2_/RbF	Spin Coating	ITO/ETL/CsMAFAPbIBr/CH_3_O-PEAI/Spiro-OMeTAD/Au	24.2 → 24.3	1.20 → 1.21	77.4 → 79.3	22.4 → 23.4	CAS, 2021 [40]
p-SnO_2_:RbF/m-SnO_2_/RbF	Spin Coating	ITO/ETL/CsMAFAPbIBrCl/Spiro-OMeTAD/Ag	23.7 → 24.5	1.11 → 1.15	77.8 → 82.1	20.6 → 22.7	Southwest Petrolium, 2022 [41]
SnO_2_/4-Imidazoleacetic acid hydrochloride (ImAcHCl)	Spin Coating	FTO/ETL/MA_0.05_FA_0.95_Pb(I_0.95_Br_0.05_)_3_/Spiro-OMeTAD/Au	22.7 → 23.1	1.09 → 1.15	79.0 → 79.0	19.5 → 21.0	SKKU, 2019 [42]
SnO_2_/KOH	CBD, Spin Coating	FTO/ETL/Cs_0.05_(FA_0.85_MA_0.15_)_0.95_Pb(I_0.85_Br_0.15_)_3_/Spiro-OMeTAD/Au	22.5 → 22.6	1.10 → 1.15	78.0 → 79.0	19.3 → 20.5	Wuhan UT, 2018 [43]
SnO_2_/Cs_2_CO_3_	Spin Coating	FTO/ETL/Cs_0.05_(MA_0.17_FA_0.83_)_0.95_Pb(I_0.83_Br_0.17_)_3_/Spiro-OMeTAD/Au	20.7 → 23.3	1.14 → 1.17	66.2 → 71.4	15.6 → 19.5	Shanghai UES, 2021 [44]
SnO_2_/KCl	Spin Coating	ITO/ETL/MAPbI_3_/Spiro-OMeTAD/Au	22.0 → 22.7	1.05 → 1.06	77.3 → 77.6	17.8 → 18.7	UST Beijing, 2020 [45]
SnO_2_/NaCl	Spin Coating	ITO/ETL/MAPbI_3_/Spiro-OMeTAD/Au	22.0 → 22.1	1.05 → 1.06	77.3 → 79.0	17.8 → 18.5	UST Beijing, 2020 [45]
SnO_2_/LiCl	Spin Coating	ITO/ETL/MAPbI_3_/Spiro-OMeTAD/Au	22.0 → 22.5	1.05 → 1.06	77.3 → 76.9	17.8 → 18.3	UST Beijing, 2020 [45]
SnO_2_/RbCl	Spin Coating	ITO/ETL/MAPbI_3_/Spiro-OMeTAD/Au	22.0 → 22.2	1.05 → 1.04	77.3 → 77.4	17.8 → 17.9	UST Beijing, 2020 [45]
SnO_2_/CsCl	Spin Coating	ITO/ETL/MAPbI_3_/Spiro-OMeTAD/Au	22.0 → 22.1	1.05 → 1.05	77.3 → 76.1	17.8 → 17.7	UST Beijing, 2020 [45]

**Table 4:** Summary of perovskite-solar cells based on bulk incorporation of ionic compounds into SnO_2_ ETL.

ETL	Method	Device Stack	*J_SC_* (mA/cm^2^)	*V_OC_* (V)	*FF* (%)	*η* (%)	Institute, Year [Ref.]
CoCl_2_:SnO_2_	Spin Coating	ITO/ETL/MAFAPbIBrCl/Spiro-OMeTAD/Au	24.2 → 24.6	1.16 → 1.20	78.9 → 80.8	22.2 → 23.8	Nankai, 2021 [46]
Heparin potassium:SnO_2_	Spin Coating	FTO/ETL/Cs_0.05_MA_0.10_FA_0.85_Pb(I_0.97_Br_0.03_)_3_/Spiro-OMeTAD/Au	24.3 → 25.0	1.13 → 1.16	75.4 → 79.4	20.7 → 23.1	Huazhong UST, 2020 [47]
KCl:SnO_2_	Spin Coating	ITO/ETL/MAFAPbIBr/Spiro-OMeTAD/Au	24.0 → 24.2	1.08 → 1.14	77.9 → 80.7	20.2 → 22.2	Nanjing, 2020 [48]
Girard’s Reagent T (GRT):SnO_2_	Spin Coating	ITO/ETL/Rb_0.05_(FA_0.95_MA_0.05_)_0.95_PbI_2.85_Br_0.15_/Spiro-OMeTAD/Au	22.6 → 22.9	1.08 → 1.15	81.2 → 82.3	19.8 → 21.6	Chongqing, 2021 [49]
NH_4_Cl:SnO_2_	Spin Coating	ITO/ETL/MAFAPbIBr/Spiro-OMeTAD/Au	23.2 → 24.3	1.10 → 1.15	73.5 → 76.8	18.7 → 21.4	Soochow, 2019 [50]
Potassium sodium tartrate (PSTA):SnO_2_	Spin Coating	ITO/ETL/MA_0.85_FA_0.15_PbI_3_/Spiro-OMeTAD/Ag	23.5 → 24.5	1.08 → 1.12	71.9 → 76.9	18.3 → 21.1	Jilin Normal, 2021 [51]
Phosphoric acid:SnO_2_	Spin Coating	ITO/ETL/MA_0.15_FA_0.85_Pb(I_0.85_Br_0.15_)_3_/Spiro-OMeTAD/Ag	22.5 → 23.2	1.18 → 1.17	73.8 → 77.4	19.7 → 21.0	CAS, 2019 [52]
Tetramethylammonium hydroxide (TMAH):SnO_2_	Spin Coating	FTO/ETL/MA_0.25_FA_0.75_PbI_2.5_Br_0.5_/Spiro-OMeTAD/Au	22.8 → 23.3	1.13 → 1.14	70.4 → 77.4	18.1 → 20.5	Shenzhen, 2018 [53]
CsF:SnO_2_	Spin Coating	FTO/ETL/Cs_0.05_(MA_0.15_FA_0.85_)_0.95_Pb(I_0.85_Br_0.15_)_3_/4-tert-butyl-D-phenylalanine (D4TBP)/Spiro-OMeTAD/Au	22.6 → 23.2	1.13 → 1.16	75.0 → 76.0	19.3 → 20.5	KMU, 2020 [54]
(NH_4_)_2_S:SnO_2_	Spin Coating	ITO/ETL/MAFAPbIBr/Spiro-OMeTAD/Ag	22.4 → 23.0	1.13 → 1.15	73.4 → 76.0	18.7 → 20.0	CAS, 2019 [55]

**Table 5:** Summary of perovskite-solar cells based on surface modification of SnO_2_ ETL by carbon materials.

ETL	Method	Device Stack	*J_SC_* (mA/cm^2^)	*V_OC_* (V)	*FF* (%)	*η* (%)	Institute, Year [Ref.]
SnO_2_/fulleropyrrolidine (NMBF-Cl)	Spin Coating	ITO/ETL/MAFAPbIBr/Spiro-OMeTAD/Ag	25.2 → 26.0	1.13 → 1.12	75.0 → 77.0	21.4 → 22.3	Wuhan UT, 2020 [56]
SnO_2_/Polystyrene (PS)	Spin Coating	ITO/ETL/MAFAPbIBr/PS/Spiro-OMeTAD/Au	23.8 → 24.0	1.09 → 1.10	74.0 → 76.0	19.3 → 20.5	CAS, 2019 [57]
SnO_2_/Graphene quantum dots	Spin Coating	ITO/ETL/MAPbI_3_/spiro-OMeTAD/Au	22.1 → 23.1	1.10 → 1.13	73.6 → 77.8	17.9 → 20.3	Zhejiang, 2017 [58]
SnO_2_/[6,6]-phenyl-C_61_-butyric acid methyl ester (PCBM)	Spin Coating	FTO/ETL/MAPbI_3_/spiro-OMeTAD/PbS/Au	22.3 → 23.3	1.13 → 1.14	75.0 → 74.0	18.8 → 19.6	Wuhan, 2017 [59]
SnO_2_/PCBM	Spin Coating	FTO/ETL/MAPbI_3_/spiro-OMeTAD/Au	21.1 → 22.6	1.09 → 1.12	71.5 → 75.8	16.5 → 19.1	Toledo, Wuhan, 2016 [60]
SnO_2_/C_60_-SAM	PEALD, Spin Coating	FTO/ETL/MAPbI_3_/spiro-OMeTAD/Au	21.2 → 21.4	1.07 → 1.13	75.5 → 79.1	17.2 → 19.0	Toledo, 2016 [61]
SnO_2_/[6,6]-phenyl-C_61_-butyric acid (PCBA)	Spin Coating	ITO/ETL/MA_0.34_FA_0.66_PbI_2.85_Br_0.15_/Spiro-OMeTAD/MoO_3_/Au	22.0 → 22.2	1.10 → 1.10	64.0 → 76.0	15.4 → 18.6	Eindhoven, 2019 [62]

**Table 6:** Summary of perovskite-solar cells based on bulk incorporation of carbon materials into SnO_2_ ETL.

ETL	Method	Device Stack	*J_SC_* (mA/cm^2^)	*V_OC_* (V)	*FF* (%)	*η* (%)	Institute, Year [Ref.]
Polymeric carbon nitrides (cPCN):SnO_2_	Spin Coating	FTO/ETL/MAFAPbIBr/Spiro-OMeTAD/Ag	23.4 → 24.9	1.11 → 1.13	82.0 → 82.5	21.3 → 23.2	CAS, 2021 [63]
Nb_2_C:SnO_2_	Spin Coating	ITO/ETL/Cs_0.05_MA_0.07_FA_0.88_PbI_3_/Spiro-OMeTAD/MoO_3_/Au	24.7 → 25.3	1.11 → 1.14	69.1 → 79.5	19.0 → 22.9	China UPB, 2021 [64]
Carbon quantum dot:SnO_2_	Spin Coating	ITO/ETL/Cs_0.05_(MA_0.17_FA_0.83_)_0.95_Pb(I_0.83_Br_0.17_)_3_/Spiro-OMeTAD/MoO_3_/Au	23.1 → 24.1	1.07 → 1.14	77.8 → 82.9	19.2 → 22.8	CAS, 2020 [65]
Polyacrylamide (PAM):SnO_2_	Spin Coating	ITO/ETL/MAFAPbIBrCl/Spiro-OMeTAD/Au	23.2 → 24.8	1.10 → 1.12	79.2 → 81.1	20.2 → 22.6	Guilin UT, CAS, 2022 [66]
g-C_3_N_4_:SnO_2_	Spin Coating	ITO/ETL/CsMAFAPbIBr/Spiro-OMeTAD/Au	23.7 → 24.0	1.11 → 1.12	76.2 → 78.3	20.2 → 22.1	Xian Jiaotong, 2020 [67]
Graphdiyne:SnO_2_	Spin Coating	ITO/ETL/CsMAFAPbIBr/Spiro-OMeTAD/Au	22.9 → 23.3	1.13 → 1.14	74.3 → 79.6	19.2 → 21.1	UST Beijing, 2020 [68]
Naphthalene diimide graphene:SnO_2_	Spin Coating	ITO/ETL/MA_0.17_FA_0.83_PbI_2.63_Br_0.37_/Spiro-OMeTAD/Au	23.2 → 22.7	1.10 → 1.08	74.6 → 82.1	19.0 → 20.2	Huazhong UST, 2018 [69]
Carbon nanodot:SnO_2_	Spin Coating	ITO/ETL/CsMAFAPbIBr/Spiro-OMeTAD/Au	22.5 → 23.1	1.08 → 1.10	76.0 → 79.0	18.5 → 20.0	UST Beijing, 2019 [70]

**Table 7:** Summary of perovskite-solar cells based on surface modification of SnO_2_ ETL by organic molecules.

ETL	Method	Device Stack	*J_SC_* (mA/cm^2^)	*V_OC_* (V)	*FF* (%)	*η* (%)	Institute, Year [Ref.]
KCl:SnO_2_/BTAC4	Spin Coating	ITO/ETL/Cs_0.05_(MA_0.15_FA_0.85_)_0.95_Pb(I_0.85_Br_0.15_)_3_/Spiro-OMeTAD/Ag	23.1 → 24.2	1.23 → 1.25	75.0 → 76.1	21.2 → 23.1	CAS, 2021 [71]
KCl:SnO_2_/Y6	Spin Coating	ITO/ETL/Cs_0.05_(MA_0.15_FA_0.85_)_0.95_Pb(I_0.85_Br_0.15_)_3_/Spiro-OMeTAD/Ag	23.1 → 23.6	1.23 → 1.24	75.0 → 75.6	21.2 → 22.1	CAS, 2021 [71]
SnO_2_/Si(OCH_3_)_3_(CH_2_)_3_I (I-SAM)	Spin Coating, Immerse in solution	ITO/ETL/Cs_0.05_(FA_0.85_MA_0.15_)_0.95_Pb(I_0.85_Br_0.15_)_3_/Spiro-OMeTAD/Au	23.0 → 23.3	1.13 → 1.19	77.4 → 77.8	20.2 → 21.4	Brown, 2021 [72]
SnO_2_/Triphenylphosphine oxide (TPPO)	Spin Coating	ITO/ETL/Cs_0.05_(FA_0.85_MA_0.15_)_0.95_Pb(I_0.85_Br_0.15_)_3_/Spiro-OMeTAD/Au	24.4 → 24.3	1.08 → 1.11	72.2 → 77.0	19.0 → 20.7	SUST, 2019 [73]
SnO_2_/Thiophene-3-acetic acid	Spin Coating	ITO/ETL/MAPbI_3_/spiro-OMeTAD/MoO_3_/Ag	22.3 → 23.0	1.07 → 1.12	73.5 → 80.1	17.5 → 20.6	South China UT, 2021 [74]
SnO_2_/Aminosulfonic acid	Spin Coating, Immerse in solution	ITO/ETL/Cs_0.05_(FA_0.85_MA_0.15_)_0.95_Pb(I_0.85_Br_0.15_)_3_/Spiro-OMeTAD/Au	21.8 → 22.8	1.12 → 1.15	74.8 → 77.9	18.2 → 20.4	Beihang, 2020 [75]
SnO_2_/p-amino benzenesulfonic acid (ABSA)	Spin Coating	ITO/ETL/MAPbI_3_/Spiro-OMeTAD/MoO_3_/Ag	22.4 → 22.9	1.10 → 1.13	73.0 → 78.8	18.0 → 20.3	South China UT, 2021 [76]
SnO_2_/Choline chloride	Spin Coating, Immerse in solution	FTO/ETL/MAPbI_3_/Spiro-OMeTAD/Au	21.3 → 22.8	1.07 → 1.15	73.9 → 72.4	16.8 → 18.9	Renmin U. China, 2020 [77]
SnO_2_/4-pyridinecarboxylic acid (PA-SAM)	Spin Coating	ITO/SnO_2_-SAM/MAPbI_3_/spiro-OMeTAD/Au	21.7 → 22.0	1.06 → 1.10	74.9 → 77.4	17.2 → 18.8	UCLA, 2017 [78]
SnO_2_/Potassium hexylxanthate	Spin Coating	ITO/ETL/MAPbI_3_/Spiro-OMeTAD/Au	21.7 → 22.6	1.03 → 1.06	73.7 → 76.9	16.6 → 18.4	Kyushu Tech., 2018 [79]
SnO_2_/4-cyanobenzoic acid (CBA-SAM)	Spin Coating	ITO/SnO_2_-SAM/MAPbI_3_/spiro-OMeTAD/Au	21.7 → 21.7	1.06 → 1.08	74.9 → 78.1	17.2 → 18.3	UCLA, 2017 [78]
SnO_2_/benzoic acid (BA-SAM)	Spin Coating	ITO/SnO_2_-SAM/MAPbI_3_/spiro-OMeTAD/Au	21.7 → 21.9	1.06 → 1.11	74.9 → 74.6	17.2 → 18.1	UCLA, 2017 [78]

**Table 8:** Summary of perovskite-solar cells based on bulk incorporation of organic molecules into SnO_2_ ETL.

ETL	Method	Device Stack	*J_SC_* (mA/cm^2^)	*V_OC_* (V)	*FF* (%)	*η* (%)	Institute, Year [Ref.]
Poly(ethylene glycol) diacrylate (PEGDA):SnO_2_	Spin Coating	ITO/ETL/FAPbI_3_/Spiro-OMeTAD/MoO_3_/Ag	24.8 → 25.3	1.09 → 1.14	80.6 → 81.0	21.8 → 23.3	CAS, Chongqing, 2021 [80]
2,2,2-trifluoroethanol:SnO_2_, O_2_ plasma	Spin Coating	ITO/ETL/MAFAPbIBr/Spiro-OMeTAD/Au	23.1 → 23.9 → 24.1	1.10 → 1.12 → 1.12	75.5 → 78.0 → 80.2	19.2 → 20.9 → 21.7	CAS, 2019 [81]
Ethylene diamine tetraacetic acid (EDTA):SnO_2_	Spin Coating	ITO/ETL/Cs_0.05_FA_0.95_PbI_3_/Spiro-OMeTAD/Au	22.8 → 24.6	1.10 → 1.11	75.5 → 79.2	18.9 → 21.6	Shaanxi Normal, 2018 [82]
Polyethylene glycol (PEG):SnO_2_	Spin Coating	ITO/ETL/Cs_0.05_FA_0.81_MA_0.14_Pb(I_0.85_Br_0.15_)_3_/Spiro-OMeTAD/Au	22.6 → 22.7	1.09 → 1.12	77.9 → 81.9	19.2 → 20.8	Peking, 2018 [83]
Polyethylenimine (PEIE):SnO_2_	Spin Coating	ITO/ETL/CsMAFAPbICl/Spiro-OMeTAD/Ag	22.9 → 23.8	1.08 → 1.14	76.0 → 76.0	18.7 → 20.6	Xidian, 2020 [84]

**Table 9:** Summary stability of ETL-modified perovskite-solar cells.

ETL	Device Stack	Encapsulated	Conditions	Continuous 1 SUN Illumination?	Duration	*η* Maintained	Institute, Year [Ref.]
F:SnO_2_	FTO/bilayer F:SnO_2_/(FAPbI_3_)_0.85_(MAPbBr_3_)_0.15_/Spiro-OMeTAD/Au	Y	40–50%, Air, RT	N	300 h	>85%	Huazhong UST, 2018 [23]
Nb:SnO_2_	FTO/Nb:SnO_2_/(FAPbI_3_)_0.85_(MAPbBr_3_)_0.15_/Spiro-OMeTAD/Au	N	Air, RT	N	288 h	90%	Shaanxi Normal U., 2017 [25]
Sb:SnO_2_	ITO/Sb:SnO_2_/MAPbI_3_/Spiro-OMeTAD/Au	N	Dessicator, RT	N	504 h	>95%	UNL, 2016 [27]
Ga:SnO_2_	AZO/Ga:SnO_2_/CsFAMAPb(Br,I)_3_/Spiro-OMeTAD/Au	N	N_2_, 1 SUN	Y	1000 h	~70%	Adolphe Merkle Inst. & HZB, 2018 [28]
Mg:SnO_2_	FTO/Mg:SnO_2_/MAPbI_3_/Spiro-OMeTAD/Au	N	<20%, Air	N	720 h	>90%	Wuhan U., 2016 [29]
In_2_O_3_/SnO_2_	ITO/ETL/MAFAPbICl/spiro-OMeTAD/Au	N	N_2_	N	1920 h	98%	Nankai, 2020 [31]
		N		Y	180 h	91%	
		N	75%	N	120 h	~80%	
SnO_2_/Eu:WO*_x_*	FTO/ETL/CsFAMAPbIBr/spiro-OMeTAD/Eu:WO*_x_*/Au	N	16–25 °C, 20–30%	Y	500 h	>90%	Jilin, 2021 [32]
		N	Ambient	N	2000 h	>90%	
TiO_2_/SnO_2_	FTO/TiO_2_-SnO_2_/MAPbI_3_/PTAA/Au	N	~20%, Air	N	1200 h	>95%	EPFL, 2017 [34]
SnO_2_/MgO	ITO/ETL/MAPbI_3_/spiro-OMeTAD/Au	N	30%	N	2568 h	67%	RTV, 2018 [35]
PbO:SnO_2/_SnO_2_	FTO/ETL/MAPbI_3_/spiro-OMeTAD/Au		RT, 15%	N	1080 h	>90%	CAS, 2021 [36]
SnO_2_/ZnO	ITO/ETL/CsPbI_2_Br/spiro-OMeTAD/MoO_3_/Ag	N	85 °C, N_2_	N	300 h	80%	South China UT, 2018 [39]
SnO_2_/RbF	ITO/ETL/CsMAFAPbIBr/CH_3_O-PEAI/Spiro-OMeTAD/Au	N	White LED light illumination	N	200 h	~75%	CAS, 2021 [40]
p-SnO_2_/RbF/m-SnO_2_/RbF	ITO/ETL/CsMAFAPbIBrCl/Spiro-OMeTAD/Ag	N	MPPT	Y	300 h	90%	Southwest Petrolium, 2022 [41]
SnO_2_/4-Imidazoleacetic acid hydrochloride (ImAcHCl)	FTO/ETL/MA_0.05_FA_0.95_Pb(I_0.95_Br_0.05_)_3_/Spiro-OMeTAD/Au	N	RT, 46–60%	N	840 h	94%	SKKU, 2019 [42]
	FTO/ETL/MA_0.05_FA_0.95_Pb(I_0.95_Br_0.05_)_3_/PTAA/Au	N	85 °C, N_2_	N	40 h	90%	SKKU, 2019 [42]
SnO_2_/Cs_2_CO_3_	FTO/ETL/Cs_0.05_(MA_0.17_FA_0.83_)_0.95_Pb(I_0.83_Br_0.17_)_3_/Spiro-OMeTAD/Au	N	35–45%	N	340 h	91%	Shanghai UES, 2021 [44]
SnO_2_/NaCl	ITO/ETL/MAPbI_3_/Spiro-OMeTAD/Au	N		N	960 h	>90%	UST Beijing, 2020 [45]
CoCl_2_:SnO_2_	ITO/ETL/MAFAPbIBrCl/Spiro-OMeTAD/Au	N	N_2_	Y	200 h	84%	Nankai, 2021 [46]
		N	60 °C, >50%	N	100 h	80%	
Heparin potassium:SnO_2_	FTO/ETL/Cs_0.05_MA_0.10_FA_0.85_Pb(I_0.97_Br_0.03_)_3_/Spiro-OMeTAD/Au	Y	60–65 °C	Y	1000 h	~97%	Huazhong UST, 2020 [47]
KCl:SnO_2_	ITO/ETL/MAFAPbIBr/Spiro-OMeTAD/Au	N		Y	120 h	88%	Nanjing, 2020 [48]
Girard’s Reagent T (GRT):SnO_2_	ITO/ETL/Rb_0.05_(FA_0.95_MA_0.05_)_0.95_PbI_2.85_Br_0.15_/Spiro-OMeTAD/Au	N	5–10%, RT	N	1440 h	96%	Chongqing, 2021 [49]
		N	60 °C, N_2_	N	720 h	100%	
		N	N_2_	Y	672 h	59%	
NH_4_Cl:SnO_2_	ITO/ETL/MAFAPbIBr/Spiro-OMeTAD/Au	N	N_2_	N	1000 h	>95%	Soochow, 2019 [50]
Potassium sodium tartrate (PSTA):SnO_2_	ITO/ETL/MA_0.85_FA_0.15_PbI_3_/Spiro-OMeTAD/Ag	N	25 °C, 45%, Air	N	1440 h	>95%	Jilin Normal, 2021 [51]
Tetramethylammonium hydroxide (TMAH):SnO_2_	FTO/ETL/MA_0.25_FA_0.75_PbI_2.5_Br_0.5_/Spiro-OMeTAD/Au	Y	15%	N	360 h	97%	Shenzhen, 2018 [53]
CsF:SnO_2_	FTO/ETL/Cs_0.05_(MA_0.15_FA_0.85_)_0.95_Pb(I_0.85_Br_0.15_)_3_/4-tert-butyl-D-phenylalanine (D4TBP)/Spiro-OMeTAD/Au	N	MPPT	Y	800 h	>90%	KMU, 2020 [54]
SnO_2_/NMBF-Cl	ITO/ETL/MAFAPbIBr/Spiro-OMeTAD/Ag	N	25–35 °C, 45–60%, Air	N	1000 h	>95%	Wuhan UT, 2020 [56]
SnO_2_/Polystyrene (PS)	ITO/ETL/MAFAPbIBr/PS/Spiro-OMeTAD/Au	N	Air	N	2800 h	>90%	CAS, 2019 [57]
		N	25 °C, 25%,MPPT	Y	72 h	>90%	
SnO_2_/GQDs	ITO/ETL/MAPbI_3_/spiro-OMeTAD/Au	N	N_2_ → 20–30%, Air	N	2160 h	>95%	Zhejiang, 2017 [58]
SnO_2_/PCBM	FTO/ETL/MAPbI_3_/spiro-OMeTAD/PbS/Au	N	Air	N	1000 h	~100%	Wuhan, 2017 [59]
SnO_2_/C_60_-SAM	FTO/ETL/MAPbI_3_/spiro-OMeTAD/Au	N	<10%, room light, N_2_	N	480 h	> 98%	Toledo, 2016 [61]
Polymeric carbon nitrides (cPCN):SnO_2_	FTO/ETL/MAFAPbIBr/Spiro-OMeTAD/Ag	N	N_2_	N	2880 h	95%	CAS, 2021 [63]
		N	25–35%, Air	N	2000 h	88%	
Nb_2_C:SnO_2_	ITO/ETL/Cs_0.05_MA_0.07_FA_0.88_PbI_3_/Spiro-OMeTAD/MoO_3_/Au	N	25 °C, 40–60%	N	960 h	98%	China UPB, 2021 [64]
Carbon quantum dot:SnO_2_	ITO/ETL/Cs_0.05_(MA_0.17_FA_0.83_)_0.95_Pb(I_0.83_Br_0.17_)_3_/Spiro-OMeTAD/MoO_3_/Au	N	25 °C, 40–60%, Air	N	1000 h	96%	CAS, 2020 [65]
Polyacrylamide (PAM):SnO_2_	ITO/ETL/MAFAPbIBrCl/Spiro-OMeTAD/Au	N	45–55%	N	1080 h	90%	Guilin UT, CAS, 2022 [66]
g-C_3_N_4_:SnO_2_	ITO/ETL/CsMAFAPbIBr/Spiro-OMeTAD/Au	N	25 °C, 60%, Air	N	1500 h	90%	Xian Jiaotong, 2020 [67]
		N	85 °C, 60%, Air	N	75 h	80%	
Carbon nanodot:SnO_2_	ITO/ETL/CsMAFAPbIBr/Spiro-OMeTAD/Au	N	20 °C, 20–30%, Air, UV	N	200 h	90%	UST Beijing, 2019 [70]
		N	Dry Air	N	1200 h	>90%	
KCl:SnO_2_/BTAC4	ITO/ETL/Cs_0.05_(MA_0.15_FA_0.85_)_0.95_Pb(I_0.85_Br_0.15_)_3_/Spiro-OMeTAD/Ag	N	35%	N	768 h	~90%	CAS, 2021 [71]
KCl:SnO_2_/Y6		N	35%	N	768 h	~90%	
SnO_2_/Si(OCH_3_)_3_(CH_2_)_3_I (I-SAM)	ITO/ETL/Cs_0.05_(FA_0.85_MA_0.15_)_0.95_Pb(I_0.85_Br_0.15_)_3_/Spiro-OMeTAD/Au	N	MPPT, N_2_, RT	Y	1200 h	>90%	Brown, 2021 [72]
SnO_2_/Thiophene-3-acetic acid	ITO/ETL/MAPbI_3_/spiro-OMeTAD/MoO_3_/Ag	N	N_2_	N	1440 h	>90%	South China UT, 2021 [74]
		N	85 °C, 70%, Air	N	130 h	>80%	
SnO_2_/Aminosulfonic acid	ITO/ETL/Cs_0.05_(FA_0.85_MA_0.15_)_0.95_Pb(I_0.85_Br_0.15_)_3_/Spiro-OMeTAD/Au	N	25–35%, Air	N	1000 h	>80%	Beihang, 2020 [75]
		N	60 °C, N_2_	N	500 h	>75%	
SnO_2_/p-amino benzenesulfonic acid (ABSA)	ITO/ETL/MAPbI_3_/Spiro-OMeTAD/MoO_3_/Ag	N	N_2_	N	720 h	57%	South China UT, 2021 [76]
SnO_2_/Potassium hexylxanthate	ITO/ETL/MAPbI_3_/Spiro-OMeTAD/Au	N	RT	N	1680 h	~90%	Kyushu Tech., 2018 [79]
Poly(ethylene glycol) diacrylate (PEGDA):SnO_2_	ITO/ETL/FAPbI_3_/Spiro-OMeTAD/MoO_3_/Ag	N	N_2_	Y	850 h	>90%	CAS, Chongqing, 2021 [80]
		N	30–35%, Air	N	1000 h	98%	
2,2,2-trifluoroethanol:SnO_2_	ITO/ETL/MAFAPbIBr/Spiro-OMeTAD/Au	N	30–40%	N	720 h	>90%	CAS, 2019 [81]
Ethylene diamine tetraacetic acid (EDTA):SnO_2_	ITO/ETL/Cs_0.05_FA_0.95_PbI_3_/Spiro-OMeTAD/Au	N	35%	N	2880 h	92%	Shaanxi Normal, 2018 [82]
		N	1 SUN	Y	120 h	86%	
Polyethylene glycol (PEG):SnO_2_	ITO/ETL/Cs_0.05_FA_0.81_MA_0.14_Pb(I_0.85_Br_0.15_)_3_/Spiro-OMeTAD/Au	N	28–35 °C, 30–80%, Air	N	2160 h	>97%	Peking, 2018 [83]
Polyethylenimine (PEIE):SnO_2_	ITO/ETL/MAFAPbI/Spiro-OMeTAD/Ag	N	40%	N	1680 h	82%	Xidian, 2020 [84]
